# Global Estimates of Prevalent and Incident Herpes Simplex Virus Type 2 Infections in 2012

**DOI:** 10.1371/journal.pone.0114989

**Published:** 2015-01-21

**Authors:** Katharine J. Looker, Amalia S. Magaret, Katherine M. E. Turner, Peter Vickerman, Sami L. Gottlieb, Lori M. Newman

**Affiliations:** 1 School of Social and Community Medicine, University of Bristol, Bristol, United Kingdom; 2 Department of Laboratory Medicine, University of Washington, Seattle, Washington, United States of America; 3 Department of Reproductive Health and Research, World Health Organization, Geneva, Switzerland; Southern Illinois University School of Medicine, United States of America

## Abstract

**Background:**

Herpes simplex virus type 2 (HSV-2) infection causes significant disease globally. Adolescent and adult infection may present as painful genital ulcers. Neonatal infection has high morbidity and mortality. Additionally, HSV-2 likely contributes substantially to the spread of HIV infection. The global burden of HSV-2 infection was last estimated for 2003. Here we present new global estimates for 2012 of the burden of prevalent (existing) and incident (new) HSV-2 infection among females and males aged 15–49 years, using updated methodology to adjust for test performance and estimate by World Health Organization (WHO) region.

**Methods and Findings:**

We conducted a literature review of HSV-2 prevalence studies world-wide since 2000. We then fitted a model with constant HSV-2 incidence by age to pooled HSV-2 prevalence values by age and sex. Prevalence values were adjusted for test sensitivity and specificity. The model estimated prevalence and incidence by sex for each WHO region to obtain global burden estimates. Uncertainty bounds were computed by refitting the model to reflect the variation in the underlying prevalence data. In 2012, we estimate that there were 417 million people aged 15–49 years (range: 274–678 million) living with HSV-2 infection world-wide (11.3% global prevalence), of whom 267 million were women. We also estimate that in 2012, 19.2 million (range: 13.0–28.6 million) individuals aged 15–49 years were newly-infected (0.5% of all individuals globally). The highest burden was in Africa. However, despite lower prevalence, South-East Asia and Western Pacific regions also contributed large numbers to the global totals because of large population sizes.

**Conclusions:**

The global burden of HSV-2 infection is large, leaving over 400 million people at increased risk of genital ulcer disease, HIV acquisition, and transmission of HSV-2 to partners or neonates. These estimates highlight the critical need for development of vaccines, microbicides, and other new HSV prevention strategies.

## Introduction

Herpes simplex virus type 2 (HSV-2) infection is mainly sexually transmitted, causing genital herpes. Infection with HSV-2 is lifelong and can be determined by serological tests that measure the presence of IgG antibodies to HSV-2 [1]. Genital herpes is characterised by periodic symptomatic or asymptomatic viral shedding [1], and causes a significant burden of disease, most notably, the periodic appearance of often painful genital ulcers and accompanying psychological morbidity in a proportion of those infected [1]. However, the clinical presentation of infection is variable, and the majority of individuals are unaware they are infected [2]. Although rare, infection in the neonate is associated with a high risk of severe morbidity and mortality [3], [4].

HSV-2 infection has received renewed attention in recent years, due to improvements in the understanding of the epidemiological synergy between HSV-2 and HIV. HSV-2 infection increases the risk of HIV acquisition by approximately three-fold [5], and the increase in risk is even greater in those with newly-acquired (incident) HSV-2 infection [6], [7]. Among those with HIV, HSV-2 infection increases genital shedding of HIV [8], increases transmissibility of HIV up to five-fold through genital ulcers [9], and may accelerate HIV disease progression [10]. In turn, HIV infection increases HSV-2 shedding frequency and quantity [11], [12]. The high burden of HSV-2 infection is thought to have contributed substantially to HIV prevalence in a number of settings, most notably in sub-Saharan Africa [5], [13], although it is difficult to control for shared sexual risk driving both epidemics [14]. Daily suppressive antiviral therapy against HSV-2 has been shown to reduce symptomatic recurrences and asymptomatic HSV shedding; however, in clinical trials, suppressive therapy did not reduce the excess risk of HIV acquisition or transmission due to HSV-2 nor fully suppress HSV-2 shedding [15]–[17].

Efforts to develop a vaccine against HSV are advancing. In clinical trials, a prophylactic vaccine failed to prevent HSV-2 infection and disease [18]. However, preliminary results from ongoing trials of post-exposure therapeutic vaccines against genital HSV-2 infection have been encouraging, demonstrating reductions in HSV-2 shedding in vaccine recipients compared to placebo [19], [20]. Further results are anticipated soon and there are other vaccines in the pipeline [21]. At a recent World Health Organization (WHO) global consultation on STI vaccine development, it was noted that to assess the potential benefit of an HSV vaccine on population-wide HSV-2 infection and ongoing HIV transmission, it is critical to quantify the burden of HSV-2 infection in populations and its distribution by age and sex [22].

The global burden of HSV-2 infection has not been evaluated since 2003 estimates were published in 2008, when we estimated for the first time that 536 million people had existing (prevalent) and 23.6 million people had new (incident) HSV-2 infection world-wide (without adjustment for test performance) [23]. This followed an earlier review of HSV seroprevalence [24] that did not pool prevalence nor estimate the global or regional burden of infection. In this analysis we present new global estimates for 2012 of the burden of prevalent and incident HSV-2 infection for females and males aged 15–49 years by WHO region, including adjustment for test performance. Such estimates are critical not only for understanding the epidemiology of HSV infection, but also for guiding development of vaccines, microbicides, diagnostics and therapeutics, and for advocating support for STI prevention and control efforts.

## Methods

The methods employed are similar to those used for the 2003 estimates [23], but with some notable refinements described in detail below. Most importantly, in these estimates adjustment was done for test sensitivity and specificity, while adjustment was not done for the 2003 estimates. In addition, for this set of estimates we applied a cut-off year of 2000 or later for inclusion of study data, while there was no cut-off for the 2003 estimates, which used data from all studies published up to the date of search in 2005. Finally, the regional groupings used differ in this set of revised estimates.

### Ethics Statement

An ethics statement was not required for this study.

### Search Strategy And Selection Criteria

PubMed and EMBASE were searched to identify potentially-relevant publications reporting HSV-2 prevalence and/or incidence published since the earlier reviews [23], [24]. MeSH terms used in the PubMed search (date of search 12/02/2014) were “seroepidemiologic studies”, “prevalence”, “cross-sectional studies”, “incidence”, “cohort studies”, “follow-up studies”, “longitudinal studies”, “time factors”, “prospective studies” OR “survival analysis”; AND: “simplexvirus”, “herpes simplex”, “herpesvirus 1, human”, “herpesvirus 2, human”, “herpes genitalis”, “herpes labialis“ OR “stomatitis, herpetic”; filters: publication date from 01/01/2005. Subject headings used in the EMBASE search (date of search 23/10/2013) were “Herpes simplex virus”, “Herpes simplex virus 1”, “Herpes simplex virus 2”, “herpes simplex”, “genital herpes” OR “herpes labialis”; AND: “seroepidemiology”, “incidence” OR “prevalence”; filters: human, publication date from 01/01/2005. No other restrictions were made, including with regard to language. Reference lists were also searched. Note that although the last set of estimates were for 2003, the review of prevalence values informing these estimates was done in 2005 and applied to 2003 population numbers.

Inclusion and exclusion criteria followed the previous reviews [23], [24] with some refinement. HSV-2 prevalence studies were eligible for inclusion if they reported the percentage of people with type-specific IgG antibodies (in blood/serum) to HSV-2 cross-sectionally, or if we could calculate this from given numbers. HSV-2 incidence studies were eligible for inclusion if they reported the rate or risk of incident detection of type-specific IgG to HSV-2 or if we could calculate this from the number of cases and reported numbers or time at risk. Prevalence values based on IgM were excluded since we were measuring established infection which is shown by the presence of IgG [25].

Studies were required to give some detail of the study location (minimum: country) and some detail of participants' age. Prevalence and incidence were extracted by sex and by age as well as overall, but not for any other characteristic. Estimated values were read from figures if exact numbers were not available. Prevalence that was weighted or adjusted to account for selection bias was used where reported. Unresolved equivocal samples were excluded from both numerator and denominator. Where comparison results from more than one test were presented, we retained results from the assay or method judged to be the most robust (e.g., Western blot, or assay plus confirmatory testing). If more than one publication presented findings from the same study, then all relevant data were extracted. However, if findings from the same subset of participants were repeated (e.g., a particular age range), then the occurrence with the largest sample size was extracted, but all relevant publications listed.

Studies were also excluded for the following reasons: 1) if study participants were selected on the basis of having a medical condition, since this may be associated with HSV infection, and the findings may not be generalisable (examples of excluded studies were studies of transplant recipients, eye infections, atherosclerosis and atopy); 2) if study individuals were selected on the basis of having a specific genital or urinary tract infection (e.g., genital ulcer disease, vaginitis, urethritis); or 3) if study individuals were selected on the basis of HSV infection and/or disease (e.g., a history of genital herpes, serodiscordant couples), since it was considered that this would bias the prevalence estimates. Studies which selected individuals on the basis of being infected with HIV were not excluded, as these individuals are a specific interest group; however these were summarised separately. Where possible, seroincidence was extracted only for individuals in the control arm of any interventions which might affect HSV incidence, and on an intention-to-treat basis.

Populations were defined as either “high-risk” or general, where general may be antenatal clinic attendees, householders or any other population not at specific high risk of infection. “High-risk” was defined as groups with particular behaviours or exposures associated with higher risk of infection, such as STI clinic attendees, men who have sex with men, commercial sex workers, injecting drug users and HIV-infected individuals. Individuals living in a high prevalence area but without behaviours which put them at specific high risk of infection were categorised as being from general populations and not “high-risk”.

### Calculation of HSV-2 Prevalence and Incidence Estimates

To estimate the numbers of people with prevalent and incident HSV-2 infection globally, only HSV-2 prevalence values from general populations and stratified by sex were used. Age-stratified prevalence values were used in preference over unstratified values. Where study participants were selected for HIV status, we used HSV-2 prevalence values for HIV-uninfected individuals (unless these individuals were “high-risk” for HSV-2 for a separate reason). In moderate to high HIV prevalence settings, using prevalence data from HIV-uninfected populations only will likely underestimate HSV-2 prevalence; however, most studies here did not select for HIV. Only publications with study year mid-point (or publication year if not known) of 2000 or later were used. Studies used in the 2003 estimates paper which were still sufficiently recent were also included. A minimum sample size of 20 was required; small sample sizes by age were combined as necessary. The mean or median age was used where reported. If prevalence was reported by age in ranges then the mid-point of the age range was taken. Sample sizes for age strata where not given were estimated from the total sample size and widths of the age strata. Age strata without finite age limits (e.g., <25 years, ≥35 years etc.) were assumed to extend for 10 years (i.e., 15–24 years, 35–44 years). Mean/median ages and mid-points of ages were then grouped as follows: 15–19; 20–24; 25–29; 30–34; 35–39; 40–44 and 45–49 years. Zero prevalence values were recoded as 0.1%.

We adjusted each prevalence value for the sensitivity and specificity of the assay used [26], according to the package insert of the assay or published test performance [27] and using the following equation:
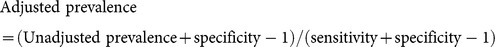
with prevalence, sensitivity and specificity expressed as proportions. Where confirmatory testing (or similar) was performed, we assumed 100% sensitivity and specificity as these values could not be known, and confirmatory testing will likely improve either or both of these values. Where the assay had unknown sensitivity or specificity, a value of 98% for each was chosen based on the range of known sensitivity and specificity values reported for the other assays. For those studies used in the 2003 estimates, we were able to re-obtain the original publications and extract the required information on type of assay used since the number of publications was small.

Prevalence was estimated separately for each of the 6 WHO regions: the Americas, Africa, Eastern Mediterranean, Europe, South-East Asia and Western Pacific, in contrast to the 2003 estimates which were done for 12 regions. A comparison of the regions used in the 2003 and 2012 estimates is shown in S1 Table. Six regions were used for the 2012 estimates to enable direct comparison with other disease burden estimates produced by WHO.

The numbers of individuals with prevalent and incident HSV-2 infection in 2012 was calculated using a similar method to the 2003 estimates [23]. For each WHO region, pooled prevalence values by sex and 5-year age group (15–49 years) were generated in Stata (Stata 13; StataCorp, College Station, Texas, USA) using the metan command to pool the raw log odds of infection weighted by the standard error of the log odds for those prevalence values with sample ages within the boundaries of each 5-year age category. A random-effect model was used for pooling, which accounts for between- as well as within-study variation.

HSV-2 incidence by sex was calibrated from these pooled prevalence values using a constant-incidence model [28]. This modelling step additionally incorporated a calibrated term for the maximum proportion able to be infected to allow prevalence to saturate at low or moderate prevalence where the pooled prevalence values indicated this (e.g., as a consequence of lower incidence at older age). The following expression explains the exponential relationship between incidence and prevalence used in the model: 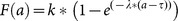
where *F*(*a*) is the proportion seropositive for HSV-2 at age *a*, *k* is the maximum proportion that can be expected to be infected over a lifetime of exposure, λ is the force of infection per year for all ages and τ is the age at which individuals are first exposed to infection (assumed τ = 12 years, which is a lower bound for commencing sexual activity). Fitting was done by using the Solver function in Excel which used maximum likelihood to find those values of *k* and λ which maximized the value of:



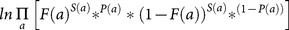
where *a* is the mid-point of each 5-year age group, *S*(*a*) is the total sample size (from summing across all studies) and *P*(*a*) is the pooled HSV-2 prevalence. By including both *k* and λ the model is able to capture observed prevalence patterns by age, including initial rapid increase in prevalence at younger ages followed by gradual levelling off of prevalence at older ages, where observed.

Once λ and *k* were computed, the smoothed HSV-2 seroprevalence estimates by sex and 5-year age group resulting from the model fits (*F*(*a*)) were then multiplied by regional population size obtained from the United Nations Population Division for 2012 [29] to estimate the numbers of people with prevalent HSV-2 infection by region in 2012. Thus, these 2012 estimates apply to the year 2012 for population size but use prevalence data from 2000 onwards, with prevalence assumed not to vary over the period. The numbers of people with incident HSV-2 infection by region in 2012 were obtained by applying the model incidence to the population sizes able to be infected. Specifically, the numbers of new cases of HSV-2 infection at each single year of age, *I*(*a*), were calculated as:
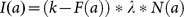
where *N*(*a*) is the total number of individuals (i.e., regional population size) at age *a*. Estimates were then summed across these ages in each 5-year age category. Model incidence was used rather than reported incidence due to a lack of reported incidence values across all ages and regions. Global estimates were obtained by summing values over all 6 regions.

An uncertainty analysis was performed for the numbers of people with prevalent and incident HSV-2 infection in 2012 as follows. Confidence intervals for the pooled age- and region-specific prevalence values from the meta-analysis describe the variation based on sample size and study-to-study population differences. For each regional set of estimates (by sex) k and λ were re-estimated from each of the lower and upper confidence bounds for the pooled prevalence values by age, to generate bounds for the regional estimates. The lower and upper bounds were summed across all regions to compute lower and upper bounds for the global estimates.

Two sensitivity analyses for the estimates were performed: first, removing adjustment for assay sensitivity and specificity, and second, assuming poorer test performance for the Focus assays (97% sensitivity and 89% specificity [30], from a default of 100% and 96%) and default sensitivity and specificity for the rest. The reason for doing the latter was that there is evidence of differing test performance by study population [30], with the performance of the Focus assay being the best studied due to it being the most frequently used. Negative and zero prevalence values generated as a consequence of adjustment (which occurs when both prevalence and specificity are low, or more accurately, when (unadjusted prevalence + specificity) ≤1) were recoded as 0.1%.

The studies used in the 2003 estimates were re-analysed with the same 6 WHO groupings, for comparison, and using 2003 population sizes from the United Nations Population Division [29]. Test adjustment was not done for the re-analysis due to the large number of studies which precluded re-obtaining the relevant publications to extract full information on type of assay used.

## Results

### Literature Search

**Figure 1:**
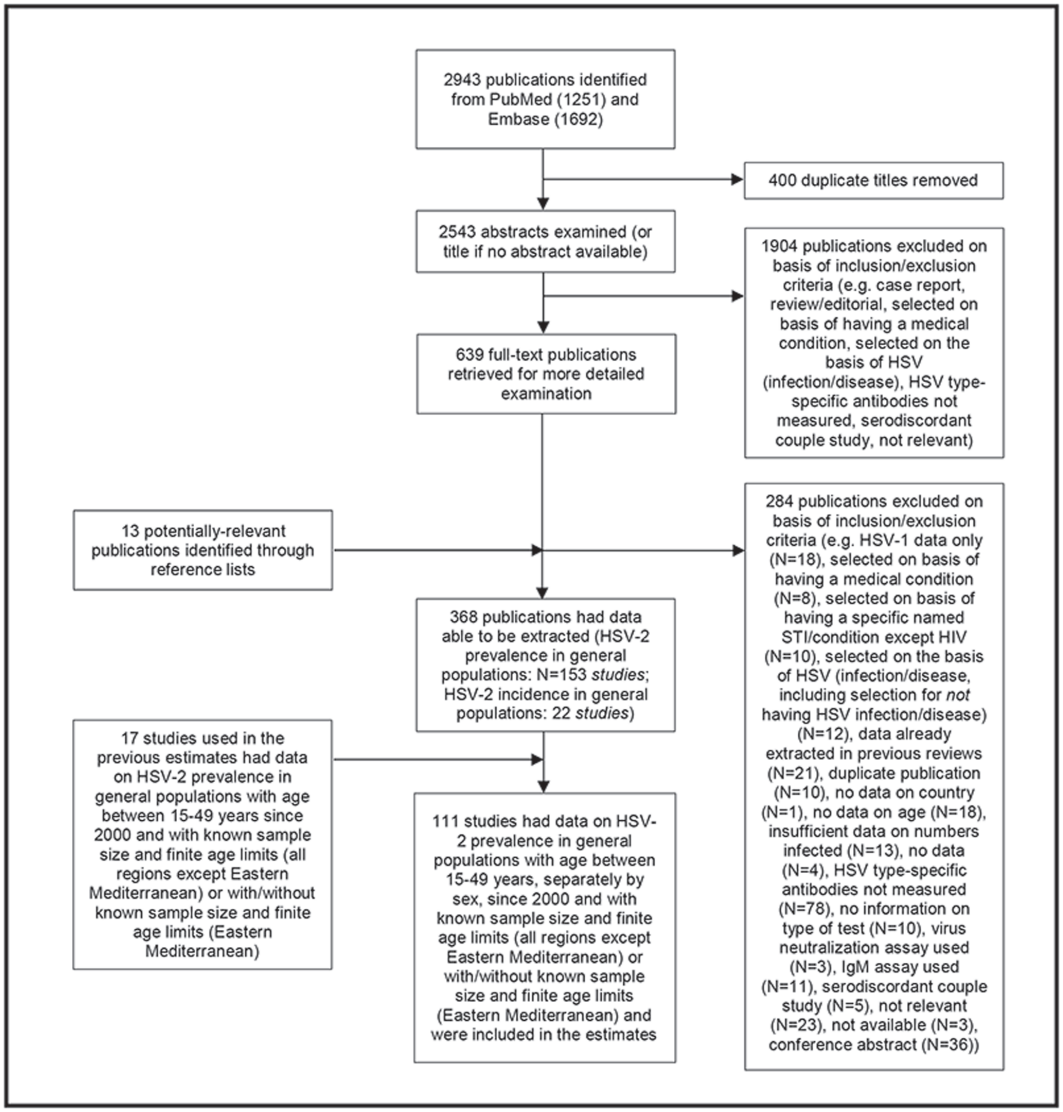
Flow chart showing selection of publications from the literature search.

A total of 2943 publications were identified: 1251 and 1692 from PubMed and Embase, respectively (Fig. 1). Removal of 400 duplicates left 2543 publications. The abstract of each (or title if not available) was examined for potential relevance, resulting in exclusion of 1904 publications on the basis of the inclusion and exclusion criteria (see Methods). This left 639 publications for which the full text was obtained. Thirteen additional potentially-relevant publications were identified through reference lists. After full-text retrieval a further 284 publications were excluded, leaving 368 publications for which data could be extracted. Of these, the number of *studies* (i.e., incorporating one or more publications) from general populations only included in the review were as follows: HSV-2 prevalence: 153 studies; HSV-2 incidence: 22 studies. Studies reporting incidence often reported baseline prevalence. Therefore studies are counted more than once where included in more than one category. (For the tables of HSV-2 prevalence and incidence in general populations from the literature review see S1 Database and S1 Reference List.)

Only studies which presented HSV-2 prevalence by sex in general populations with age within the range 15–49 years since 2000 were used in the estimates. A further limitation was that only HSV-2 prevalence values with known sample size and finite age limits were included. The exception was for Eastern Mediterranean where no such restrictions were made due to limited available data. This left 94 studies which provided data for the estimates. A further 17 studies used in the 2003 estimates [23] met the same criteria, resulting in a total of 111 studies (S1 Database; the prevalence observations included in the estimates are indicated). A summary of the numbers of studies used to compute prevalence by region, sex and 5-year age group, and the individual countries for which data were available is shown in S2 Table. Some regions were heavily influenced by only one or two countries. For example, the United States of America and Canada together contributed two-thirds of prevalence values used to calculate the Americas estimates, India contributed all but two prevalence values used to calculate the South-East Asia estimates, and China and the Republic of Korea together contributed >80% of prevalence values used to calculate the Western Pacific estimates. However, India, China and the United States of America also comprise a large proportion of their regional population size. Note that studies are often represented more than once since many studies reported prevalence for more than one age group. S3 Table shows the frequency of assays used, and the corresponding sensitivity and specificity values applied to estimate prevalence. Focus was by far the most common assay used, followed by Kalon and Western blot.

### Prevalent HSV-2 Infection in 2012

**Figure 2:**
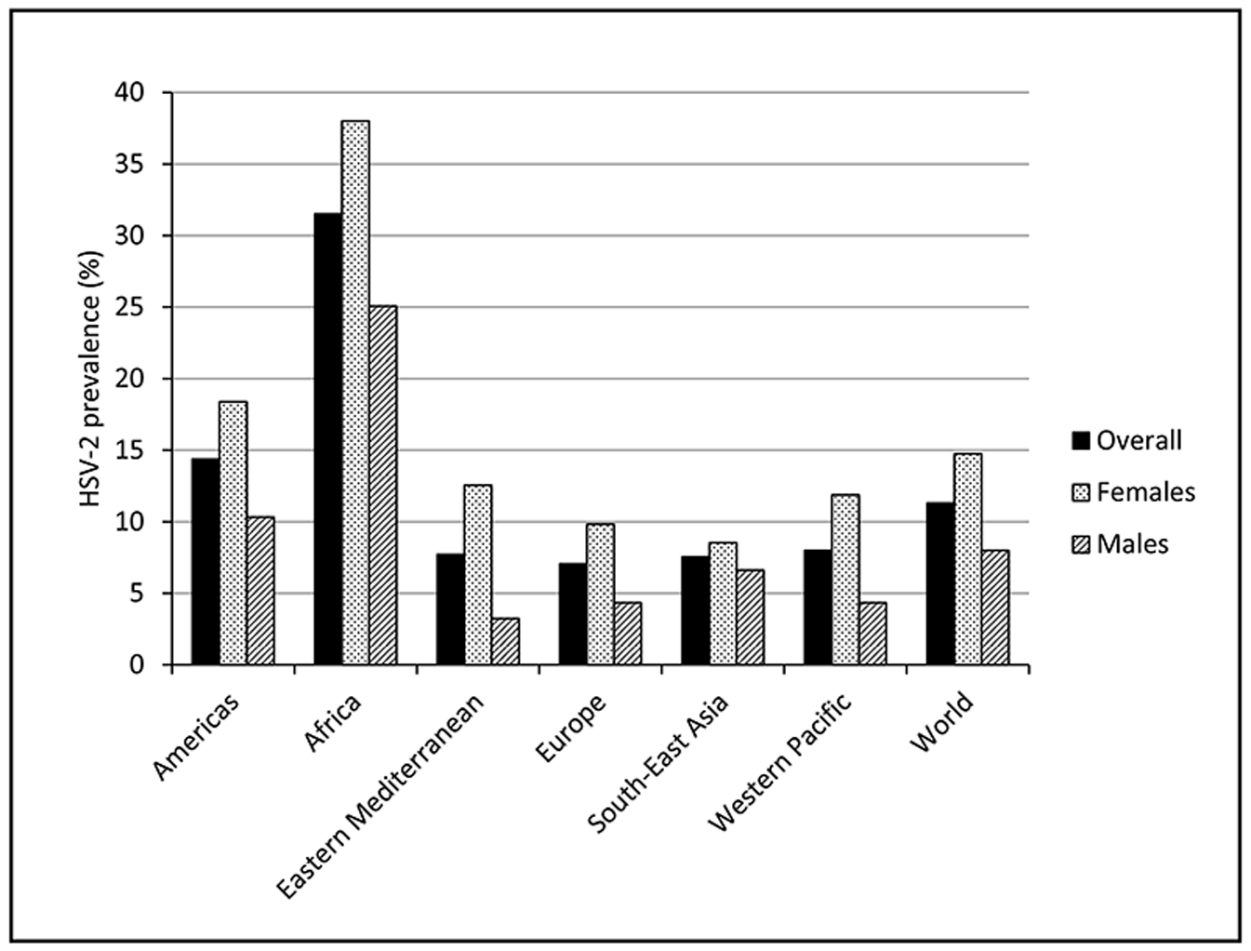
Estimated HSV-2 prevalence among 15–49 year olds in 2012 resulting from the model fits.

**Figure 3:**
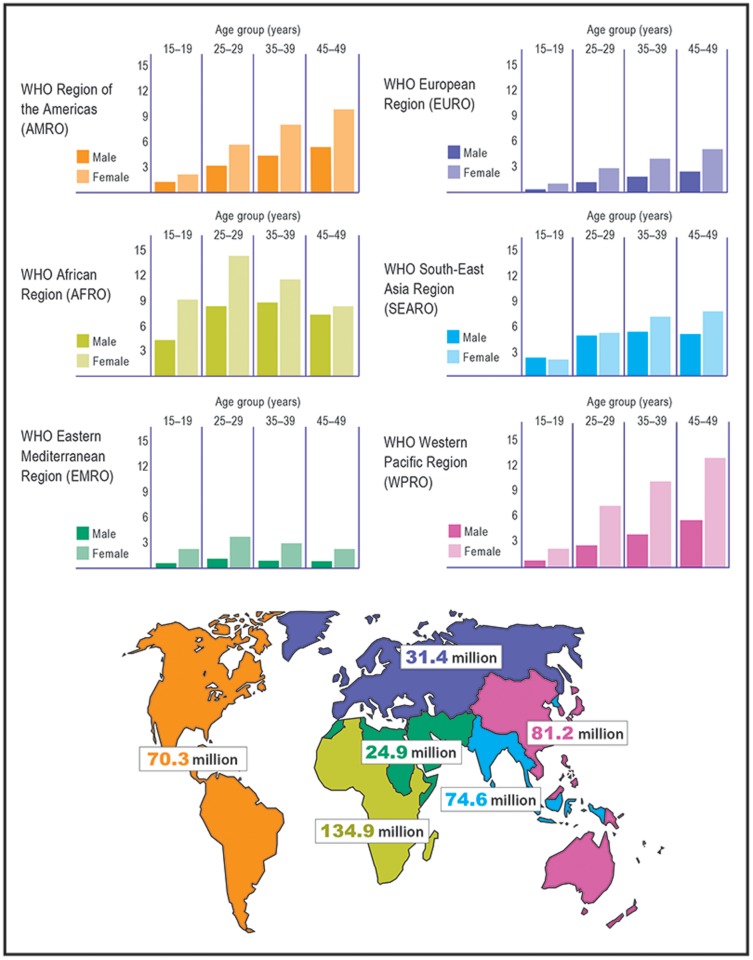
Estimates of the number of people (in millions) with prevalent HSV-2 infection in 2012, by age, sex and WHO region. Note that only selected age groups are shown for simplicity.

**Figure 4:**
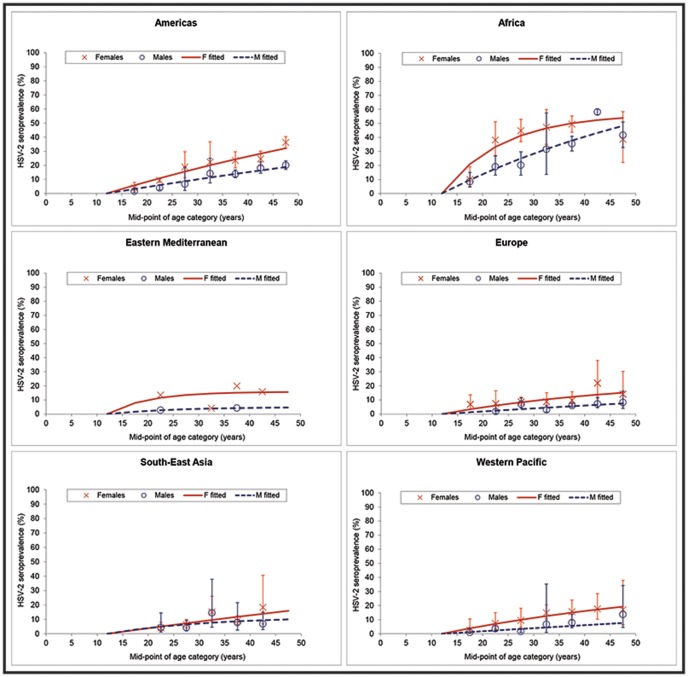
Model fits to pooled HSV-2 prevalence values by age and sex, by WHO region. Confidence intervals are not shown for the pooled prevalence values for Eastern Mediterranean since these included data without finite age limits and/or with estimated sample size.

The overall prevalence of HSV-2 among 15–49 year olds world-wide in 2012 is estimated to be 11.3% from the model fits (Fig. 2). Prevalence was highest in Africa (31.5%) followed by the Americas (14.4%), and consistently higher in females compared to males (global prevalence in females and males: 14.8% versus 8.0%) (Fig. 2). Using these modelled prevalence values, the total number of people aged 15–49 years world-wide with prevalent HSV-2 infection in 2012 is estimated to be 417 million (Table 1). More women (267 million) than men (150 million) were infected, and both the prevalence and number infected increased with age, especially in the younger age groups (Table 1). Similar patterns were seen by region (Figs. 3 and 4; Table 1). Africa and the Americas contributed large numbers infected to the global totals to reflect their underlying higher prevalence. However, South-East Asia and Western Pacific also contributed large numbers infected to the global totals by virtue of their large population sizes relative to other regions despite moderate prevalence. Declines in the numbers infected at older ages by region, where seen (Fig. 3: Africa, females and males; Eastern Mediterranean, females and males; South-East Asia, males) are due to the underlying population structure, particularly for those regions with growing population, in most cases combined with prevalence saturating (“levelling out”) in the model fits (i.e., slow or no increase in prevalence at older ages) (Fig. 4). For detailed results from the meta-analysis see S4 Table.

**Table 1:** Global and regional estimates of the number of existing (prevalent) cases of HSV-2 infection in 2012 by age and sex, in millions (percentage of population with prevalent infection in each age group shown in parentheses).

Both								
	Age group (years)							
	15–19	20–24	25–29^a^	30–34	35–39^a^	40–44	45–49	All ages
**Global total (all)** a	**27.6 (4.6%)**	**48.2 (7.8%)**	**60.9 (10.5%)**	**65.7 (12.7%)**	**69.6 (14.3%)**	**72.8 (15.6%)**	**72.7 (17.0%)**	**417.3 (11.3%)**

Females								
Region	Age group (years)							
	15–19	20–24	25–29	30–34	35–39	40–44^a^	45–49	All ages^a^
**Americas** a	2.1	4.0	5.6	6.9	8.0	8.9	9.8	**45.2**
**Africa** a	9.1	13.3	14.6	13.8	11.9	10.0	8.5	**81.1**
**Eastern Mediterranean**	2.1	3.2	3.6	3.3	2.8	2.4	2.1	**19.5**
**Europe** a	0.8	1.8	2.7	3.3	3.8	4.3	4.9	**21.7**
**South-East Asia**	2.0	3.8	5.4	6.6	7.4	7.9	8.0	**41.1**
**Western Pacific**	2.2	5.3	7.3	7.7	10.2	12.9	13.0	**58.6**
**Global total (females)**	**18.3**	**31.4**	**39.2**	**41.6**	**44.1**	**46.3**	**46.3**	**267.3 (14.8%)**

Males								
Region	Age group (years)							
	15–19	20–24	25–29^a^	30–34	35–39^a^	40–44	45–49	All ages
**Americas** a	1.2	2.2	3.2	3.9	4.4	4.9	5.4	**25.1**
**Africa** a	4.2	7.1	8.8	9.3	9.0	8.2	7.3	**53.8**
**Eastern Mediterranean** a	0.5	0.8	1.0	1.0	0.9	0.7	0.7	**5.4**
**Europe** a	0.3	0.7	1.1	1.4	1.7	2.0	2.3	**9.7**
**South-East Asia**	2.3	4.0	5.1	5.6	5.7	5.6	5.2	**33.5**
**Western Pacific** a	0.8	1.9	2.6	2.8	3.9	5.1	5.4	**22.6**
**Global total (males)** a	**9.3**	**16.7**	**21.7**	**24.0**	**25.5**	**26.5**	**26.3**	**150.1 (8.0%)**

a Totals slightly different due to rounding.

### Incident HSV-2 Infection in 2012

Using the calibrated model incidence, the total number of people aged 15–49 years with incident HSV-2 infection in 2012 is estimated to be 19.2 million: 11.8 million women and 7.4 million men (Table 2). The numbers newly-infected with HSV-2 are highest in the younger age groups and decline with increased age as the number of uninfected individuals able to be infected decreases. Similar results were seen by region (Table 2). The decline in the number newly-infected with age was particularly marked for some regions as a consequence of the underlying population structure, and especially due to saturation in prevalence in some model fits (Fig. 4). It is not certain whether or not prevalence does reach saturation in settings with low or moderate prevalence (e.g., Eastern Mediterranean), or whether saturation in the modelled prevalence, or indeed absence of saturation in other model fits, is an artefact due to lack of data supporting the model fits. In theory, age-related changes in risk behaviour over time (cohort effects) or differential mortality due to AIDS could enable prevalence to saturate at older ages and even decline, however these are not modelled here.

**Table 2:** Global and regional estimates of the number of new (incident) cases of HSV-2 infection in 2012 by age and sex, in millions (percentage of population with incident infection in each age group shown in parentheses).

Both								
	Age group (years)							
	15–19	20–24	25–29	30–34	35–39	40–44	45–49	All ages
**Global total (all)**	**4.9 (0.8%)**	**3.9 (0.6%)**	**3.1 (0.5%)**	**2.3 (0.5%)**	**1.9 (0.4%)**	**1.7 (0.4%)**	**1.4 (0.3%)**	**19.2 (0.5%)**

Females								
Region	Age group (years)							
	15–19	20–24	25–29	30–34	35–39	40–44	45–49	All ages^a^
**Americas**	0.401	0.375	0.346	0.310	0.277	0.250	0.230	**2.2**
**Africa**	1.490	0.859	0.491	0.268	0.141	0.074	0.040	**3.4**
**Eastern Mediterranean** a	0.315	0.164	0.081	0.036	0.015	0.007	0.003	**0.6**
**Europe**	0.156	0.159	0.147	0.126	0.108	0.094	0.085	**0.9**
**South-East Asia**	0.393	0.373	0.349	0.316	0.277	0.244	0.210	**2.2**
**Western Pacific**	0.422	0.485	0.421	0.319	0.324	0.323	0.267	**2.6**
**Global total (females)** a	**3.2**	**2.4**	**1.8**	**1.4**	**1.1**	**1.0**	**0.8**	**11.8 (0.6%)**

Males								
Region	Age group (years)							
	15–19	20–24	25–29	30–34	35–39	40–44	45–49	All ages^a^
**Americas** a	0.229	0.216	0.201	0.182	0.163	0.148	0.139	**1.3**
**Africa** a	0.813	0.644	0.507	0.384	0.282	0.203	0.148	**3.0**
**Eastern Mediterranean**	0.078	0.054	0.036	0.022	0.012	0.007	0.004	**0.2**
**Europe** a	0.064	0.071	0.073	0.068	0.065	0.062	0.061	**0.5**
**South-East Asia**	0.417	0.309	0.227	0.163	0.114	0.080	0.055	**1.4**
**Western Pacific**	0.155	0.189	0.173	0.139	0.152	0.165	0.148	**1.1**
**Global total (males)** a	**1.8**	**1.5**	**1.2**	**1.0**	**0.8**	**0.7**	**0.6**	**7.4 (0.4%)**

a Totals slightly different due to rounding.

### Uncertainty Analysis

Refitting the model to the lower and upper bounds of the pooled prevalence values generates uncertainty bounds of 274–678 million prevalent HSV-2 infections globally: 184–395 million women and 89–283 million men with prevalent HSV-2 infection (S5 Table). The global number of incident HSV-2 infections, meanwhile, is calculated to be in the range 13.0–28.6 million (8.3–16.5 million women and 4.7–12.1 million men with incident HSV-2 infection) (S5 Table). These wide intervals reflect the large uncertainty in the prevalence data informing the estimates calculations, both due to sampling variability, which is magnified by small number of studies and small sample sizes, and as a consequence of differences in populations across the same region.

### Adjustment for Assay Type

The total numbers with new and existing HSV-2 infection in 2012 are sensitive to the adjustments made for type of assay used. The total number of people with prevalent HSV-2 infection is higher without adjustment (518 million) and lower when lower sensitivity and specificity values for Focus are applied (306 million) (S6 Table). This is because correction for decreased specificity results in a lower prevalence while correction for decreased sensitivity results in a higher prevalence. Correcting for low sensitivity will have a smaller impact when prevalence is low while correcting for low specificity will have a greater impact; thus, most estimates will be revised downwards by adjustment for test performance. The lower test performance for Focus used in the sensitivity analysis resulted in almost half of the prevalence values using Focus being adjusted below zero before recoding and is unlikely to be realistic.

### Comparison with 2003 Estimates

Data availability was similar between the 2003 and 2012 estimates, although there was some difference in which countries were represented. Of particular note, India and China were represented proportionally more in the data informing the 2012 estimates. Re-analysis of the 2003 data by the 6 WHO regions gives an estimate for the total number of people aged 15–49 years with prevalent HSV-2 infection globally in 2003 of 507 million (Table 3), in contrast to the estimate of 536 million previously obtained when analysing the data by 12 regions. The number newly-infected with HSV-2 in 2003, meanwhile, is estimated to be 21.1 million from the 6 region re-analysis, compared to the estimate of 23.6 million obtained using the 12 region groupings (Table 3). Note that adjustment for type of assay used was not done for any of these 2003 estimates, and that our analysis of the 2012 estimates suggests that with test adjustment the estimated burden of infection would be lowered.

**Table 3:** Comparison between 2003 and 2012 global and regional estimates of the number of existing (prevalent) and new (incident) cases of HSV-2 infection by sex, in millions (percentage of total population with prevalent/incident infection shown in parentheses).

Region	2003				2012			
	Prevalent infection		Incident infection		Prevalent infection		Incident infection	
	Females^a^	Males	Females	Males	Females	Males	Females^a^	Males
**Americas**	57.3	29.2	2.3	1.5	49.8	30.6	2.4	1.5
**Africa**	79.3	46.6	3.5	2.6	81.8	54.0	3.3	3.0
**Eastern Mediterranean**	17.3	16.3	0.7	0.3	23.7	16.1	0.6	0.2
**Europe**	34.9	15.2	0.9	0.8	27.3	15.1	0.9	0.5
**South-East Asia**	52.9	46.1	2.8	0.8	54.8	47.5	2.6	1.5
**Western Pacific**	54.9	56.5	2.1	2.8	73.1	43.9	2.7	2.1
**Global total**	**296.7 (18.1%)**	**209.9 (12.4%)**	**12.3 (0.8%)**	**8.8 (0.5%)**	**310.5 (17.1%)**	**207.2 (11.0%)**	**12.4 (0.7%)**	**8.8 (0.5%)**
**Global total (all)**	**506.6 (15.2%)**		**21.1 (0.6%)**		**517.7 (14.0%)**		**21.2 (0.6%)**	

a Totals slightly different due to rounding. Note that test adjustment was not done for these estimates.

Since test adjustment was not possible in the re-analysis of the 2003 data, the most appropriate comparison between the estimates years is to compare the re-analysed 2003 estimates with the 2012 estimates with no test adjustment (Table 3). Using this methodology, the number of individuals with prevalent HSV-2 infection world-wide was 507 million in 2003 and 518 million in 2012 estimates. If these estimates represent a true increase in the numbers of people with HSV infection, this would be due to increasing population size, as overall prevalence was estimated to be 15.2% in 2003 and 14.0% in 2012. However, estimates based on small numbers of studies can generate substantial random variation between estimates years, which in turn impacts on the model fits. For example, there was substantial variation in reported prevalence across all ages for females in Africa (S4 Table). Furthermore, the countries included are not always the same between estimates. Therefore, interpreting prevalence trends over time using these estimates, which are generally not based on repeated surveys in the same populations, would not be advised.

## Discussion

We estimate that in 2012 there were 417 million people (range: 274–678 million) (11.3%) world-wide aged 15–49 years with existing HSV-2 infection and that 19.2 million (range: 13.0–28.6 million) became newly infected with HSV-2. Since HSV-2 infection is lifelong, most of these prevalent infections will be in the same people from year to year. This contrasts with, for example, at any one time in 2008, 100 million people with *Chlamydia trachomatis* infection (106 million newly-infected), 36 million with *Neisseria gonorrhoeae* infection (106 million newly-infected) and 36 million with syphilis (11 million newly-infected) world-wide [31].

More women than men had prevalent infection (267 million versus 150 million). The most likely reason for this is that women have greater biological susceptibility to HSV-2 [32]–[35], although different patterns of sexual behaviour between the sexes (such as different patterns of sexual mixing) might expose women to a higher risk of infection. HSV-2 prevalence increases with age, as infection is lifelong. Trends in prevalence over time can confound trends over age. In these 2012 estimates we used only recently-collected data (i.e., since 2000), which will lessen the influence of cohort effects on the age-specific prevalence of infection, and also by extension, minimise the likelihood of there being time trends within the time period for included studies.

Patterns of infection by region were very different. Africa had the highest prevalence (32%), followed by the Americas (14%), with Africa contributing most to the global totals due to combined large population and high prevalence. Despite their lower prevalence, South-East Asia (8%) and Western Pacific (8%) also contributed large numbers infected to the global totals due to their large population numbers. Similar patterns in HSV-2 infection by sex, age and region were found in the 2003 estimates, and are consistent with a literature review from 2001 [24]. Model incidence was used rather than reported values due to a lack of studies; however, the fitted model incidences were in line with published values (S1 Database).

The numbers of adults infected with HSV-2 was computed slightly higher in the 2012 estimates compared with the 2003 estimates. This was largely due to greater population size in 2012, as the computed global prevalence underlying the estimates was slightly lower in 2012. However since global prevalence is a product of relative regional population size and is therefore influenced by the relative growth rates of high versus low prevalence countries, trends in global prevalence are more difficult to interpret than regional trends. Furthermore, there may be spuriously high or low prevalence values included between estimates years, giving rise to erroneous time trends. In the USA, the National Health and Nutrition Examination Survey (NHANES) has since 1976 regularly examined the seroprevalence of HSV-1 and HSV-2 in a number of repeated nationally-representative surveys. Thus, we can reliably say that HSV-2 prevalence in the USA increased between study years 1976–1980 and 1988–1994, decreased between study years 1988–1994 and 1999–2004, and has plateaued since [36], [37]. However, for other regions there are no equivalent, nationally-representative surveys done across a number of study years, and we do not even have the same countries providing data between the 2003 and 2012 estimates. Variation in the underlying prevalence data is so large as to envelope any possible trend in global prevalence over time, as shown by the wide uncertainty bounds around the estimates. Thus, the most we can say is that the global burden of HSV-2 has remained high since 2003.

### Limitations

The accuracy of the estimates of the global burden of HSV-2 infection is limited by poor availability of data, and small sample sizes for those data that are available. For the Eastern Mediterranean region, available data were too limited to generate an estimate with satisfactory confidence, which highlights the need for more data for this region [38]. Data availability was similar between the 2003 and 2012 estimates. Since the 2003 estimates were done, several countries have published prevalence values for the first time, but often only in selected “high-risk” groups which were not used here. For other countries, surveys in general populations have not been updated in over a decade and are becoming out-of-date. In some cases HSV-2 prevalence could not be included in our estimates due to lack of information on the participants' age, type of assay used or sample size, or because the numbers were incomplete.

Potentially, additional data could have been obtained by broadening the search terms in PubMed to include text terms (rather than using only MeSH terms), and by including conference abstracts and grey literature. However, broadening the search terms would have vastly increased the number of non-relevant records retrieved and the reviewing time, with likely little additional gain in terms of useable data. For conference abstracts and grey literature, there was concern about the inclusion of data which had not been subject to rigorous peer review. A further limitation is the variable quality of those data that were available. We did not exclude any study on the basis of study quality, except to exclude those studies which did not measure IgG antibodies to HSV-2 using a type-specific assay, and which did not provide sufficient study information. We attempted to control for test performance by adjusting prevalence values for the sensitivity and specificity of the assay used to derive them. However, while an improvement, the adjustment is not complete, and some variability and uncertainty remain. We did not specifically account for the choice of assay cut-off in the assay sensitivity and specificity. For example, some studies using the Focus assay used a higher cut-off than that recommended by the manufacturer, to try to improve assay specificity [30]. However, it is unclear whether low cut-off positives, which would be counted as positives using the recommended cut-off, but counted as negatives using the higher cut-off, are true negatives or whether they represent early seroconversions [39], [40]. Additionally, there is evidence that assays might perform differently in different settings, either geographically or between different population groups [30], although this is not well understood. We did not specifically account for this as this is an evolving area of research. However, we made additional adjustments to account for potential variability in the sensitivity and specificity of the Focus assay. We found that the estimates are sensitive to the type of adjustment done, although patterns remain unchanged.

Studies may not be generalizable to an entire country, and even less so to a whole region. By using fewer regions, the estimates should be more robust against the influence of individual studies since the number of studies informing each regional set of estimates is greater. However, it also follows that by grouping a greater number of countries together, there could be greater within-region heterogeneity, and generalizability could potentially become even more of an issue. Weighted or adjusted rates were used where reported, which should improve the generalizability of findings. However this was done in only a minority of studies. The wide bounds around the estimates are reflective of the combined issues of limited data availability, small sample sizes and potential for heterogeneity in prevalence across regions. More good-quality, general population prevalence surveys across a number of different countries and settings in each region are required to obtain accurate and robust estimates of the burden of infection across each region.

### Additional Considerations

Our estimates represent only the burden of HSV-2 infection, and not HSV-2-related disease. However, if we conservatively assume that 10–20% of genital HSV-2 infections are symptomatic [2], [37], [41], then an estimated 40–80 million people around the world may suffer from painful recurrent genital ulceration in any one year. Further, all HSV-2 infections can potentially contribute to the spread of HIV infection. Presence of HSV-2 infection, as measured by type-specific antibodies, is associated with an increased risk of HIV acquisition, even in the absence of recognized symptoms of genital ulcer disease [5].

In addition, our estimates do not consider the contribution of infection by HSV type 1 (HSV-1), which can also cause genital herpes [1]. Historically, transmission routes have been divided: sexual contact for HSV-2 infection, and oral-to-oral contact for HSV-1 infection causing oro-labial herpes. However, there is an emerging trend of increasing genital HSV-1 infection among young people in many settings [42]–[46]. This is likely a consequence of decreases in childhood oral HSV-1 infection and hence lowered childhood-acquired HSV-1 immunity [37], together with an increase in rates of oral sex following sexual maturity [47]. Although type-specific serological tests can measure the presence of HSV-1 antibodies, they are unable to determine the site of infection [48]. Recurrences due to genital HSV-1 infection are much less frequent than with genital HSV-2 infection [49], and globally most symptomatic genital herpes is due to HSV-2 [1]. However, studies suggest that the risk of transmission to the neonate is greater for a maternal incident infection due to genital HSV-1 than for HSV-2 in mothers shedding HSV at delivery [50]. Future estimates could be expanded to look at the burden of HSV-1 infection; however, disentangling the separate contributions of oral versus genital HSV-1 transmission routes to HSV-1 incidence and prevalence requires careful consideration. Future estimates could also be done in “high-risk” populations to analyse the burden of infection in specific at-risk groups.

### Impact and Recommendations

These new estimates represent the best attempt to date to measure HSV-2 infection globally, incorporating adjustment for assay characteristics and a cut-off year for inclusion of prevalence studies. Our finding that approximately 417 million people world-wide have HSV-2 infection reinforces findings from previous assessments that the global burden of HSV-2 infection remains high. In light of the role of HSV-2 infection in enhancing HIV acquisition and transmission, these estimates emphasize the need to continue pursuing strategies to prevent HSV infection. HSV-2 infection is also an important infection in its own right. It is potentially fatal when transmitted to neonates, and causes distressing symptoms in a substantial number of adolescents and adults: our estimates suggest that well over 40 million people suffer from recurrent HSV genital ulcer disease.

Through these estimates, we hope to increase understanding and awareness of this highly prevalent infection, enable more accurate estimation of its contribution to onward HIV transmission, and facilitate modelling of the potential impact of future prevention efforts. Indeed, with progress being made in the development of new vaccines against either HSV-2 infection or disease, these new estimates of the burden of HSV-2 infection are highly timely. Precision regarding the burden of infection is critical information for HSV vaccine development and future vaccine implementation and evaluation efforts [22], development of other primary prevention methods such as microbicides, and new treatments and diagnostics. A better understanding of HSV-2 infection in different populations will enable more accurate estimation of the potential for HSV-2 vaccines of varying efficacy, microbicides and other interventions to reduce the burden of HSV-2 and its subsequent impact on HIV infection. In addition, precise estimates by region are crucial for guiding broader STI prevention and control efforts.

## Supporting Information

S1 Table **Comparison of region groupings between the 2003 and 2012 estimates.**
(DOCX)

S2 Table **Number of studies contributing HSV-2 prevalence in general populations to the 2012 estimates, by region.**
(DOCX)

S3 Table **Test adjustor (sensitivity and specificity) values used, by assay type.**
(DOCX)

S4 Table **Pooled log odds of infection, τ^2^ values and I^2^ values, from the meta-analysis.**
(DOCX)

S5 Table **Lower and upper bounds for the global and regional estimates of the number of existing (prevalent) and new (incident) cases of HSV-2 infection in 2012 by sex, in millions, as a function of uncertainty in the underlying HSV-2 prevalence data.**
(DOCX)

S6 Table **Sensitivity analysis for the global estimates of the number of existing (prevalent) cases of HSV-2 infection in 2012 by age, in millions (percentage of population with prevalent infection in each age group shown in parentheses), as a function of test adjustment.**
(DOCX)

S1 Database **Tables of HSV-2 prevalence and incidence in general populations from the current literature review, and included from previous reviews.**
(XLSX)

S1 Reference List **List of publications to accompany** **S1 Database** **.**
(DOCX)

S1 Checklist **The PRISMA Checklist used in this study.**
(DOC)
