# Correction: Global Estimates of Prevalent and Incident Herpes Simplex Virus Type 2 Infections in 2012

**DOI:** 10.1371/journal.pone.0128615

**Published:** 2015-05-20

**Authors:** Katharine J. Looker, Amalia S. Magaret, Katherine M. E. Turner, Peter Vickerman, Sami L. Gottlieb, Lori M. Newman

The following information is missing from the Funding section: This study was also supported by NIH grant P01-A1-030731-23 (to ASM).
