# Supplementary material for: Global Estimates of Prevalent and Incident Herpes Simplex Virus Type 2 Infections in 2012
Source: PLoS One. 2015 Jan 21;10(1):e114989. doi: 10.1371/journal.pone.0114989 (PMC4301914; doi:10.1371/journal.pone.0114989)
Supplement: S1 Table — Comparison of region groupings between the 2003 and 2012 estimates. (DOCX) [file pone.0114989.s001.docx]

**Table S1** Comparison of region groupings between the 2003 and 2012 estimates

| **Country name** | **2012 region** | **2003 region** |
| --- | --- | --- |
| Algeria | Africa | Middle East and North Africa |
| Angola | Africa | Sub-Saharan Africa |
| Benin | Africa | Sub-Saharan Africa |
| Botswana | Africa | Sub-Saharan Africa |
| Burkina Faso | Africa | Sub-Saharan Africa |
| Burundi | Africa | Sub-Saharan Africa |
| Cameroon | Africa | Sub-Saharan Africa |
| Cape Verde | Africa | Sub-Saharan Africa |
| Central African Republic | Africa | Sub-Saharan Africa |
| Chad | Africa | Sub-Saharan Africa |
| Comoros | Africa | Sub-Saharan Africa |
| Congo | Africa | Sub-Saharan Africa |
| Côte d'Ivoire | Africa | Sub-Saharan Africa |
| Democratic Republic of the Congo | Africa | Sub-Saharan Africa |
| Equatorial Guinea | Africa | Sub-Saharan Africa |
| Eritrea | Africa | Sub-Saharan Africa |
| Ethiopia | Africa | Sub-Saharan Africa |
| Gabon | Africa | Sub-Saharan Africa |
| Gambia | Africa | Sub-Saharan Africa |
| Ghana | Africa | Sub-Saharan Africa |
| Guinea | Africa | Sub-Saharan Africa |
| Guinea-Bissau | Africa | Sub-Saharan Africa |
| Kenya | Africa | Sub-Saharan Africa |
| Lesotho | Africa | Sub-Saharan Africa |
| Liberia | Africa | Sub-Saharan Africa |
| Madagascar | Africa | Sub-Saharan Africa |
| Malawi | Africa | Sub-Saharan Africa |
| Mali | Africa | Sub-Saharan Africa |
| Mauritania | Africa | Sub-Saharan Africa |
| Mauritius | Africa | Sub-Saharan Africa |
| Mozambique | Africa | Sub-Saharan Africa |
| Namibia | Africa | Sub-Saharan Africa |
| Niger | Africa | Sub-Saharan Africa |
| Nigeria | Africa | Sub-Saharan Africa |
| Rwanda | Africa | Sub-Saharan Africa |
| Sao Tome and Principe | Africa | Sub-Saharan Africa |
| Senegal | Africa | Sub-Saharan Africa |
| Seychelles | Africa | Sub-Saharan Africa |
| Sierra Leone | Africa | Sub-Saharan Africa |
| South Africa | Africa | Sub-Saharan Africa |
| South Sudan | Africa | -- |
| Swaziland | Africa | Sub-Saharan Africa |
| Togo | Africa | Sub-Saharan Africa |
| Uganda | Africa | Sub-Saharan Africa |
| United Republic of Tanzania | Africa | Sub-Saharan Africa |
| Zambia | Africa | Sub-Saharan Africa |
| Zimbabwe | Africa | Sub-Saharan Africa |
| Antigua and Barbuda | Americas | Latin America and Caribbean |
| Argentina | Americas | Latin America and Caribbean |
| Bahamas | Americas | Latin America and Caribbean |
| Barbados | Americas | Latin America and Caribbean |
| Belize | Americas | Latin America and Caribbean |
| Bolivia | Americas | Latin America and Caribbean |
| Brazil | Americas | Latin America and Caribbean |
| Canada | Americas | North America |
| Chile | Americas | Latin America and Caribbean |
| Colombia | Americas | Latin America and Caribbean |
| Costa Rica | Americas | Latin America and Caribbean |
| Cuba | Americas | Latin America and Caribbean |
| Dominica | Americas | Latin America and Caribbean |
| Dominican Republic | Americas | Latin America and Caribbean |
| Ecuador | Americas | Latin America and Caribbean |
| El Salvador | Americas | Latin America and Caribbean |
| Grenada | Americas | Latin America and Caribbean |
| Guatemala | Americas | Latin America and Caribbean |
| Guyana | Americas | Latin America and Caribbean |
| Haiti | Americas | Latin America and Caribbean |
| Honduras | Americas | Latin America and Caribbean |
| Jamaica | Americas | Latin America and Caribbean |
| Mexico | Americas | Latin America and Caribbean |
| Nicaragua | Americas | Latin America and Caribbean |
| Panama | Americas | Latin America and Caribbean |
| Paraguay | Americas | Latin America and Caribbean |
| Peru | Americas | Latin America and Caribbean |
| Saint Kitts and Nevis | Americas | Latin America and Caribbean |
| Saint Lucia | Americas | Latin America and Caribbean |
| Saint Vincent and the Grenadines | Americas | Latin America and Caribbean |
| Suriname | Americas | Latin America and Caribbean |
| Trinidad and Tobago | Americas | Latin America and Caribbean |
| United States of America | Americas | North America |
| Uruguay | Americas | Latin America and Caribbean |
| Venezuela (Bolivarian Republic of) | Americas | Latin America and Caribbean |
| Afghanistan | Eastern Mediterranean | South Asia |
| Bahrain | Eastern Mediterranean | Middle East and North Africa |
| Djibouti | Eastern Mediterranean | Middle East and North Africa |
| Egypt | Eastern Mediterranean | Middle East and North Africa |
| Iran (Islamic Republic of) | Eastern Mediterranean | Middle East and North Africa |
| Iraq | Eastern Mediterranean | Middle East and North Africa |
| Jordan | Eastern Mediterranean | Middle East and North Africa |
| Kuwait | Eastern Mediterranean | Middle East and North Africa |
| Lebanon | Eastern Mediterranean | Middle East and North Africa |
| Libyan Arab Jamahiriya | Eastern Mediterranean | Middle East and North Africa |
| Morocco | Eastern Mediterranean | Middle East and North Africa |
| Oman | Eastern Mediterranean | Middle East and North Africa |
| Pakistan | Eastern Mediterranean | South Asia |
| Qatar | Eastern Mediterranean | Middle East and North Africa |
| Saudi Arabia | Eastern Mediterranean | Middle East and North Africa |
| Somalia | Eastern Mediterranean | Sub-Saharan Africa |
| Sudan | Eastern Mediterranean | Sub-Saharan Africa |
| Syrian Arab Republic | Eastern Mediterranean | Middle East and North Africa |
| Tunisia | Eastern Mediterranean | Middle East and North Africa |
| United Arab Emirates | Eastern Mediterranean | Middle East and North Africa |
| Yemen | Eastern Mediterranean | Middle East and North Africa |
| Albania | Europe | Europe and Central Asia |
| Andorra | Europe | Western Europe |
| Armenia | Europe | Europe and Central Asia |
| Austria | Europe | Western Europe |
| Azerbaijan | Europe | Europe and Central Asia |
| Belarus | Europe | Europe and Central Asia |
| Belgium | Europe | Western Europe |
| Bosnia and Herzegovina | Europe | Europe and Central Asia |
| Bulgaria | Europe | Europe and Central Asia |
| Croatia | Europe | Europe and Central Asia |
| Cyprus | Europe | Middle East and North Africa |
| Czech Republic | Europe | Europe and Central Asia |
| Denmark | Europe | Western Europe |
| Estonia | Europe | Europe and Central Asia |
| Finland | Europe | Western Europe |
| France | Europe | Western Europe |
| Georgia | Europe | Europe and Central Asia |
| Germany | Europe | Western Europe |
| Greece | Europe | Western Europe |
| Hungary | Europe | Europe and Central Asia |
| Iceland | Europe | Western Europe |
| Ireland | Europe | Western Europe |
| Israel | Europe | Middle East and North Africa |
| Italy | Europe | Western Europe |
| Kazakhstan | Europe | Europe and Central Asia |
| Kyrgyzstan | Europe | Europe and Central Asia |
| Latvia | Europe | Europe and Central Asia |
| Lithuania | Europe | Europe and Central Asia |
| Luxembourg | Europe | Western Europe |
| Malta | Europe | Middle East and North Africa |
| Monaco | Europe | Western Europe |
| Montenegro | Europe | -- |
| Netherlands | Europe | Western Europe |
| Norway | Europe | Western Europe |
| Poland | Europe | Europe and Central Asia |
| Portugal | Europe | Western Europe |
| Republic of Moldova | Europe | Europe and Central Asia |
| Romania | Europe | Europe and Central Asia |
| Russian Federation | Europe | Europe and Central Asia |
| San Marino | Europe | Western Europe |
| Serbia | Europe | -- |
| Slovakia | Europe | Europe and Central Asia |
| Slovenia | Europe | Western Europe |
| Spain | Europe | Western Europe |
| Sweden | Europe | Western Europe |
| Switzerland | Europe | Western Europe |
| Tajikistan | Europe | Europe and Central Asia |
| The former Yugoslav Republic of Macedonia | Europe | Europe and Central Asia |
| Turkey | Europe | Europe and Central Asia |
| Turkmenistan | Europe | Europe and Central Asia |
| Ukraine | Europe | Europe and Central Asia |
| United Kingdom of Great Britain and Northern Ireland | Europe | Western Europe |
| Uzbekistan | Europe | Europe and Central Asia |
| Bangladesh | South-East Asia | South Asia |
| Bhutan | South-East Asia | South Asia |
| Democratic People's Republic of Korea | South-East Asia | Eastern Asia |
| India | South-East Asia | South Asia |
| Indonesia | South-East Asia | South-eastern Asia |
| Maldives | South-East Asia | South Asia |
| Myanmar | South-East Asia | South-eastern Asia |
| Nepal | South-East Asia | South Asia |
| Sri Lanka | South-East Asia | South Asia |
| Thailand | South-East Asia | South-eastern Asia |
| Timor-Leste | South-East Asia | South-eastern Asia |
| Australia | Western Pacific | Australia & New Zealand |
| Brunei Darussalam | Western Pacific | Eastern Asia |
| Cambodia | Western Pacific | South-eastern Asia |
| China | Western Pacific | Eastern Asia |
| Cook Islands | Western Pacific | Pacific |
| Fiji | Western Pacific | Pacific |
| Japan | Western Pacific | Japan |
| Kiribati | Western Pacific | Pacific |
| Lao People's Democratic Republic | Western Pacific | South-eastern Asia |
| Malaysia | Western Pacific | South-eastern Asia |
| Marshall Islands | Western Pacific | Pacific |
| Micronesia (Federated States of) | Western Pacific | Pacific |
| Mongolia | Western Pacific | Eastern Asia |
| Nauru | Western Pacific | Pacific |
| New Zealand | Western Pacific | Australia & New Zealand |
| Niue | Western Pacific | Pacific |
| Palau | Western Pacific | Pacific |
| Papua New Guinea | Western Pacific | Pacific |
| Philippines | Western Pacific | South-eastern Asia |
| Republic of Korea | Western Pacific | Eastern Asia |
| Samoa | Western Pacific | Pacific |
| Singapore | Western Pacific | Eastern Asia |
| Solomon Islands | Western Pacific | Pacific |
| Tonga | Western Pacific | Pacific |
| Tuvalu | Western Pacific | Pacific |
| Vanuatu | Western Pacific | Pacific |
| Viet Nam | Western Pacific | South-eastern Asia |
| Serbia and Montenegro | -- | Europe and Central Asia |
