# Supplementary material for: Global Estimates of Prevalent and Incident Herpes Simplex Virus Type 2 Infections in 2012
Source: PLoS One. 2015 Jan 21;10(1):e114989. doi: 10.1371/journal.pone.0114989 (PMC4301914; doi:10.1371/journal.pone.0114989)
Supplement: S2 Table — Number of studies contributing HSV-2 prevalence in general populations to the 2012 estimates, by region. (DOCX) [file pone.0114989.s002.docx]

**Table S2** Number of studies contributing HSV-2 prevalence in general populations to the 2012 estimates, by region

| **Region** | **Sex** | **Number of studies contributing to estimate** | | | | | | | **% of world population in region by sex^d^** | **Countries included** |
| --- | --- | --- | --- | --- | --- | --- | --- | --- | --- | --- |
|  |  | **15-19 years** | **20-24 years** | **25-29 years** | **30-34 years** | **35-39 years** | **40-44 years** | **45-49 years** |  |  |
| **Americas^a^** | F | 12 | 16 | 10 | 6 | 7 | 5 | 4 | 13.6 | Brazil, Canada, Columbia, Honduras, Mexico, Peru and United States of America |
|  | M | 9 | 12 | 8 | 8 | 5 | 3 | 4 | 12.9 | Brazil, Canada, Honduras, Mexico, Peru and United States of America |
| **Africa^a^** | F | 9 | 14 | 19 | 9 | 8 | 1^c^ | 4 | 11.8 | Benin, Burkina Faso, Gabon, Kenya, Malawi, Nigeria, Rwanda, South Africa, Uganda, United Republic of Tanzania, Zambia and Zimbabwe |
|  | M | 5 | 14 | 7 | 3 | 7 | 2 | 3 | 11.4 | Benin, Burkina Faso, Kenya, Malawi, Nigeria, Rwanda, South Africa, Uganda, United Republic of Tanzania and Zimbabwe |
| **Eastern Mediterranean^b^** | F | 1^c^ | 4 | 1^c^ | 3 | 2 | 3 | 0 | 8.6 | Iran (Islamic Republic of), Jordan and Morocco |
|  | M | 1^c^ | 5 | 1^c^ | 1^c^ | 2 | 1^c^ | 0 | 8.9 | Afghanistan, Iran (Islamic Republic of), Jordan, Morocco and Pakistan |
| **Europe^a^** | F | 2 | 12 | 13 | 14 | 6 | 6 | 3 | 12.2 | Belgium, Croatia, Finland, France, Germany, Israel, Italy, Poland, Russian Federation, Serbia, Sweden, Switzerland, The Netherlands and Turkey |
|  | M | 1^c^ | 5 | 4 | 5 | 4 | 2 | 2 | 11.9 | Croatia, Germany, Poland, Russian Federation and Turkey |
| **South-East Asia^a^** | F | 1^c^ | 6 | 6 | 4 | 4 | 3 | 1^c^ | 26.6 | India and Thailand |
|  | M | 1^c^ | 4 | 4 | 5 | 2 | 3 | 1^c^ | 27.0 | Democratic Republic of Timor-Leste and India |
| **Western Pacific^a^** | F | 3 | 7 | 8 | 7 | 5 | 2 | 3 | 27.2 | Australia, China, Japan, Papua New Guinea and Republic of Korea |
|  | M | 3 | 4 | 5 | 4 | 3 | 1^c^ | 3 | 27.8 | China, Japan, Papua New Guinea and Republic of Korea |

^a^Studies with finite age limits and known sample size only; ^b^All studies included; ^c^Not used in calculation of estimates since N=1; **^d^**Percentages for males don’t total 100% due to rounding. Table format adapted from [[1](#_ENREF_1)].

1. Abrahams, N., et al., *World-wide prevalence of non-partner sexual violence: a systematic review.* Lancet, 2014.
