# Supplementary material for: Global Estimates of Prevalent and Incident Herpes Simplex Virus Type 2 Infections in 2012
Source: PLoS One. 2015 Jan 21;10(1):e114989. doi: 10.1371/journal.pone.0114989 (PMC4301914; doi:10.1371/journal.pone.0114989)
Supplement: S3 Table — Test adjustor (sensitivity and specificity) values used, by assay type. (DOCX) [file pone.0114989.s003.docx]

**Table S3** Test adjustor (sensitivity and specificity) values used, by assay type

| **Manufacturer** | **Assay name** | **Number of prevalence values using assay** | **Sensitivity (%) according to product insert/published data** | **Specificity (%) according to product insert/published data** |
| --- | --- | --- | --- | --- |
| **Adaltis** | EIAgen | 3 | 98 | 98 |
| **Biokit USA** | Biokit (POCKit) | 4 | 96 | 98 |
| **DIA-Pro** | HSV1&2 IgG | 19 | 98 | 98 |
| **Euroimmun US** | HSV2 ELISA IgG | 8 | 93 | 100 |
| **Focus Diagnostics** | HerpeSelect2 | 185 | 100 (97^b^[[1](#_ENREF_1)]) | 96 (89^b^[[1](#_ENREF_1)]) |
| **Generic** | -- | 59 | Unknown^a^ | Unknown^a^ |
| **Gull (now Meridian Biosciences)** | HSV gG-2 | 4 | 81 | 99 |
| **Hope Laboratories** | HSV 1/2 IgG | 4 | 100 | 100 |
| **Kalon Biological Ltd** | HSV type 2 IgG | 60 | 96 | 99 |
| **Novum (now DRG Diagnostics)** | HSV type 2 IgG | 4 | Unknown^a^ | Unknown^a^ |
| **Radim** | Herpes IgG | 9 | 98 | Unknown^a^ |
| **United Bioresearch Inc.** | HSV Type II IgG | 2 | Unknown^a^ | 100 |
| **Vircell Microbiologists** | HSV2 IgG/IgM | 4 | 98 | 89 |
| **--** | Western blot | 56 | 100 | 100 |

Only studies with known sample size and finite age limits included, except for Eastern Mediterranean, where no such restrictions were made. ^a^Value of 98% assumed; ^b^Used in sensitivity analysis.

1. Ashley-Morrow, R., et al., *Performance of focus ELISA tests for herpes simplex virus type 1 (HSV-1) and HSV-2 antibodies among women in ten diverse geographical locations.* Clin Microbiol Infect, 2004. **10**(6): p. 530-6.
