# Supplementary material for: Global Estimates of Prevalent and Incident Herpes Simplex Virus Type 2 Infections in 2012
Source: PLoS One. 2015 Jan 21;10(1):e114989. doi: 10.1371/journal.pone.0114989 (PMC4301914; doi:10.1371/journal.pone.0114989)
Supplement: S4 Table — Pooled log odds of infection, τ2 values and I2 values, from the meta-analysis. (DOCX) [file pone.0114989.s004.docx]

**Table S4** Pooled log odds of infection, τ^2^ values and I^2^ values, from the meta-analysis

| **Americas** | | | | | | | | | |
| --- | --- | --- | --- | --- | --- | --- | --- | --- | --- |
| **Females** | | | | | **Males** | | | | |
| **Age group (years)** | **N** | **Pooled log odds of infection** | **τ^2^** | **I^2^** | **Age group (years)** | **N** | **Pooled log odds of infection** | **τ^2^** | **I^2^** |
| **15-19** | 12 | -3.17 | 1.19 | 91.2% | **15-19** | 9 | -4.14 | 1.44 | 85.9% |
| **20-24** | 16 | -2.28 | 0.06 | 66.6% | **20-24** | 12 | -3.19 | 0.47 | 87.9% |
| **25-29** | 10 | -1.47 | 0.87 | 97.9% | **25-29** | 8 | -2.64 | 2.49 | 97.9% |
| **30-34** | 6 | -1.25 | 0.70 | 97.4% | **30-34** | 8 | -1.81 | 1.02 | 98.8% |
| **35-39** | 7 | -1.18 | 0.15 | 91.7% | **35-39** | 5 | -1.83 | 0.04 | 64.0% |
| **40-44** | 5 | -1.12 | 0.07 | 75.9% | **40-44** | 3 | -1.51 | 0.03 | 64.1% |
| **45-49** | 4 | -0.56 | 0.02 | 72.5% | **45-49** | 4 | -1.39 | 0.02 | 62.6% |
| **Africa** | | | | | | | | | |
| **Females** | | | | | **Males** | | | | |
| **Age group (years)** | **N** | **Pooled log odds of infection** | **τ^2^** | **I^2^** | **Age group (years)** | **N** | **Pooled log odds of infection** | **τ^2^** | **I^2^** |
| **15-19** | 9 | -2.20 | 1.17 | 97.9% | **15-19** | 5 | -2.38 | 0.50 | 97.8% |
| **20-24** | 14 | -0.48 | 0.98 | 99.0% | **20-24** | 14 | -1.45 | 0.70 | 98.6% |
| **25-29** | 19 | -0.21 | 0.50 | 98.8% | **25-29** | 7 | -1.38 | 0.41 | 96.9% |
| **30-34** | 9 | -0.11 | 0.56 | 97.9% | **30-34** | 3 | -0.77 | 0.86 | 96.9% |
| **35-39** | 8 | -0.02 | 0.10 | 95.5% | **35-39** | 7 | -0.60 | 0.08 | 92.7% |
| **40-44** | 1 | -- | -- | -- | **40-44** | 2 | 0.34 | 0.00 | 0.0% |
| **45-49** | 4 | -0.46 | 0.60 | 94.1% | **45-49** | 3 | -0.34 | 0.07 | 66.3% |
| **Eastern Mediterranean** | | | | | | | | | |
| **Females** | | | | | **Males** | | | | |
| **Age group (years)** | **N** | **Pooled log odds of infection** | **τ^2^** | **I^2^** | **Age group (years)** | **N** | **Pooled log odds of infection** | **τ^2^** | **I^2^** |
| **15-19** | 1 | -- | -- | -- | **15-19** | 1 | -- | -- | -- |
| **20-24** | 4 | -1.87 | 1.07 | 96.3% | **20-24** | 5 | -3.60 | 5.98 | 98.8% |
| **25-29** | 1 | -- | -- | -- | **25-29** | 1 | -- | -- | -- |
| **30-34** | 3 | -3.15 | 1.82 | 93.5% | **30-34** | 1 | -- | -- | -- |
| **35-39** | 2 | -1.40 | 0.36 | 93.9% | **35-39** | 2 | -3.13 | 1.04 | 95.0% |
| **40-44** | 3 | -1.68 | 0.36 | 88.1% | **40-44** | 1 | -- | -- | -- |
| **45-49** | 0 | -- | -- | -- | **45-49** | 0 | -- | -- | -- |
| **Europe** | | | | | | | | | |
| **Females** | | | | | **Males** | | | | |
| **Age group (years)** | **N** | **Pooled log odds of infection** | **τ^2^** | **I^2^** | **Age group (years)** | **N** | **Pooled log odds of infection** | **τ^2^** | **I^2^** |
| **15-19** | 2 | -2.61 | 0.00 | 0.0% | **15-19** | 1 | -- | -- | -- |
| **20-24** | 12 | -2.54 | 2.22 | 96.5% | **20-24** | 5 | -4.00 | 1.23 | 66.2% |
| **25-29** | 13 | -2.37 | 0.30 | 78.5% | **25-29** | 4 | -2.60 | 0.13 | 44.8% |
| **30-34** | 14 | -2.35 | 1.21 | 96.5% | **30-34** | 5 | -3.51 | 0.59 | 76.3% |
| **35-39** | 6 | -2.23 | 0.30 | 65.3% | **35-39** | 4 | -2.77 | 0.00 | 0.0% |
| **40-44** | 6 | -1.27 | 0.86 | 93.4% | **40-44** | 2 | -2.56 | 0.00 | 0.0% |
| **45-49** | 3 | -1.81 | 0.62 | 85.6% | **45-49** | 2 | -2.41 | 0.12 | 35.8% |
| **South-East Asia** | | | | | | | | | |
| **Females** | | | | | **Males** | | | | |
| **Age group (years)** | **N** | **Pooled log odds of infection** | **τ^2^** | **I^2^** | **Age group (years)** | **N** | **Pooled log odds of infection** | **τ^2^** | **I^2^** |
| **15-19** | 1 | -- | -- | -- | **15-19** | 1 | -- | -- | -- |
| **20-24** | 6 | -3.16 | 0.54 | 89.9% | **20-24** | 4 | -3.11 | 1.56 | 89.4% |
| **25-29** | 6 | -2.68 | 0.28 | 89.8% | **25-29** | 4 | -3.10 | 0.53 | 89.0% |
| **30-34** | 4 | -1.73 | 0.36 | 88.0% | **30-34** | 5 | -1.77 | 2.01 | 96.2% |
| **35-39** | 4 | -2.31 | 0.09 | 71.1% | **35-39** | 2 | -2.47 | 0.69 | 94.9% |
| **40-44** | 3 | -1.50 | 0.91 | 94.5% | **40-44** | 3 | -2.62 | 0.52 | 88.8% |
| **45-49** | 1 | -- | -- | -- | **45-49** | 1 | -- | -- | -- |
| **Western Pacific** | | | | | | | | | |
| **Females** | | | | | **Males** | | | | |
| **Age group (years)** | **N** | **Pooled log odds of infection** | **τ^2^** | **I^2^** | **Age group (years)** | **N** | **Pooled log odds of infection** | **τ^2^** | **I^2^** |
| **15-19** | 3 | -3.62 | 0.73 | 41.2% | **15-19** | 3 | -4.67 | 0.00 | 0.0% |
| **20-24** | 7 | -2.52 | 0.89 | 90.5% | **20-24** | 4 | -3.32 | 0.00 | 0.0% |
| **25-29** | 8 | -2.26 | 1.12 | 98.0% | **25-29** | 5 | -4.10 | 0.32 | 66.7% |
| **30-34** | 7 | -1.74 | 0.19 | 78.4% | **30-34** | 4 | -2.63 | 4.06 | 97.1% |
| **35-39** | 5 | -1.69 | 0.32 | 87.7% | **35-39** | 3 | -2.46 | 0.19 | 62.9% |
| **40-44** | 2 | -1.55 | 0.10 | 43.5% | **40-44** | 1 | -- | -- | -- |
| **45-49** | 3 | -1.59 | 0.87 | 94.0% | **45-49** | 3 | -1.85 | 1.03 | 93.6% |

N: number of observations; N≥2 required for pooling; τ^2^: measure of between-study variance in log odds; I^2^: percentage of variation in study log odds due to between-study variation.
