# Supplementary material for: Global Estimates of Prevalent and Incident Herpes Simplex Virus Type 2 Infections in 2012
Source: PLoS One. 2015 Jan 21;10(1):e114989. doi: 10.1371/journal.pone.0114989 (PMC4301914; doi:10.1371/journal.pone.0114989)
Supplement: S5 Table — Lower and upper bounds for the global and regional estimates of the number of existing (prevalent) and new (incident) cases of HSV-2 infection in 2012 by sex, in millions, as a function of uncertainty in the underlying HSV-2 prevalence data. (DOCX) [file pone.0114989.s005.docx]

**Table S5** Lower and upper bounds for the global and regional estimates of the number of existing (prevalent) and new (incident) cases of HSV-2 infection in 2012 by sex, in millions, as a function of uncertainty in the underlying HSV-2 prevalence data

| **Both** | | | | |
| --- | --- | --- | --- | --- |
|  | **Prevalent infection** | | **Incident infection** | |
|  | **Lower bound** | **Upper bound** | **Lower bound** | **Upper bound** |
| **Global total (all)** | **273.5 (7.4%)** | **678.0 (18.4%)** | **13.0 (0.4%)** | **28.6 (0.8%)** |
| **Females** | | | | |
| **Region** | **Prevalent infection** | | **Incident infection** | |
|  | **Lower bound** | **Upper bound** | **Lower bound** | **Upper bound** |
| **Americas** | 33.6 (13.7%) | 60.5 (24.6%) | 1.661 (0.7%) | 2.715 (1.1%) |
| **Africa** | 64.0 (30.0%) | 100.5 (47.1%) | 2.864 (1.3%) | 3.755 (1.8%) |
| **Eastern Mediterranean** | 19.5 (12.6%)**^b^** | 19.5 (12.6%)**^b^** | 0.623 (0.4%)**^b^** | 0.623 (0.4%)**^b^** |
| **Europe** | 11.5 (5.2%) | 39.4 (17.8%) | 0.410 (0.2%) | 1.675 (0.8%) |
| **South-East Asia** | 23.1 (4.8%) | 71.6 (14.8%) | 1.232 (0.3%) | 3.666 (0.8%) |
| **Western Pacific** | 32.3 (6.6%) | 103.3 (20.9%) | 1.509 (0.3%) | 4.106 (0.8%) |
| **Global total (females)** | **184.1^a^ (10.2%)** | **394.7^a^ (21.8%)** | **8.3 (0.5%)** | **16.5 (0.9%)** |
| **Males** | | | | |
| **Region** | **Prevalent infection** | | **Incident infection** | |
|  | **Lower bound** | **Upper bound** | **Lower bound** | **Upper bound** |
| **Americas** | 16.3 (6.7%) | 39.7 (16.3%) | 0.844 (0.3%) | 1.884 (0.8%) |
| **Africa** | 40.9 (19.1%) | 69.9 (32.6%) | 2.328 (1.1%) | 3.370 (1.6%) |
| **Eastern Mediterranean** | 5.4 (3.2%)**^b^** | 5.4 (3.2%)**^b^** | 0.213 (0.1%)**^b^** | 0.213 (0.1%)**^b^** |
| **Europe** | 6.1 (2.8%) | 16.3 (7.3%) | 0.298 (0.1%) | 0.366 (0.2%) |
| **South-East Asia** | 12.1 (2.4%) | 86.3 (17.0%) | 0.647 (0.1%) | 3.082 (0.6%) |
| **Western Pacific** | 8.5 (1.6%) | 65.6 (12.5%) | 0.420 (0.1%) | 3.145 (0.6%) |
| **Global total (males)** | **89.4^a^ (4.8%)** | **283.3^a^ (15.1%)** | **4.7^a^ (0.3%)** | **12.1 (0.6%)** |

Lower and upper bounds for each regional set of estimates (by sex) were computed by refitting the constant-incidence model firstly to the lower bounds of the pooled prevalence values by age, and then to the upper bounds of the pooled prevalence values by age. These were then separately summed over all regions to give lower and upper bounds for the global estimates. **^a^**Totals slightly different due to rounding; ^b^Uncertainty analysis not done to inclusion of prevalence values with estimated sample size and/or undefined age limits (default estimates used).
