# Supplementary material for: Global Estimates of Prevalent and Incident Herpes Simplex Virus Type 2 Infections in 2012
Source: PLoS One. 2015 Jan 21;10(1):e114989. doi: 10.1371/journal.pone.0114989 (PMC4301914; doi:10.1371/journal.pone.0114989)
Supplement: S6 Table — Sensitivity analysis for the global estimates of the number of existing (prevalent) cases of HSV-2 infection in 2012 by age, in millions (percentage of population with prevalent infection in each age group shown in parentheses), as a function of test adjustment. (DOCX) [file pone.0114989.s006.docx]

**Table S6** Sensitivity analysis for the global estimates of the number of existing (prevalent) cases of HSV-2 infection in 2012 by age, in millions (percentage of population with prevalent infection in each age group shown in parentheses), as a function of test adjustment

| **Age (years)** | **Default assay sensitivity and specificity values^a^** | **No adjustment for sensitivity or specificity^a^** | **Lower sensitivity and specificity values for Focus^a^** |
| --- | --- | --- | --- |
| **15-19** | 27.6 (4.6%) | 37.8 (6.4%) | 23.1 (3.9%) |
| **20-24** | 48.2 (7.8%) | 63.3 (10.3%) | 37.3 (6.1%) |
| **25-29** | 60.9 (10.5%) | 77.4 (13.3%) | 45.6 (7.8%) |
| **30-34** | 65.7 (12.7%) | 81.0 (15.7%) | 48.3 (9.4%) |
| **35-39** | 69.6 (14.3%) | 84.7 (17.4%) | 49.8 (10.2%) |
| **40-44** | 72.8 (15.6%) | 87.6 (18.7%) | 50.9 (10.9%) |
| **45-49** | 72.7 (17.0%) | 85.9 (20.1%) | 50.8 (11.9%) |
| **Global total (all)** | **417.3 (11.3%)** | **517.7 (14.0%)** | **305.7 (8.3%)** |

**^a^**Totals slightly different due to rounding.
