# Supplementary material for: Global Estimates of Prevalent and Incident Herpes Simplex Virus Type 2 Infections in 2012
Source: PLoS One. 2015 Jan 21;10(1):e114989. doi: 10.1371/journal.pone.0114989 (PMC4301914; doi:10.1371/journal.pone.0114989)
Supplement: S1 Reference List — List of publications to accompany S1 Database . (DOCX) [file pone.0114989.s008.docx]

**Reference List S1** List of publications to accompany Database S1

1. Anuradha, K., et al., Herpes simplex virus 2 infection: a risk factor for HIV infection in heterosexuals. Indian J Dermatol Venereol Leprol, 2008. 74(3): p. 230-3.

2. Anvikar, A.R., et al., Seroprevalence of sexually transmitted viruses in the tribal population of Central India. Int J Infect Dis, 2009. 13(1): p. 37-9.

3. Arora, P., et al., HIV, HSV-2 and syphilis among married couples in India: patterns of discordance and concordance. Sex Transm Infect, 2011. 87(6): p. 516-20.

4. Bautista, C.T., et al., Herpes simplex virus type 2 and HIV infection among US military personnel: implications for health prevention programmes. Int J STD AIDS, 2009. 20(9): p. 634-7.

5. Becker, M.L., et al., Prevalence and determinants of HIV infection in South India: a heterogeneous, rural epidemic. AIDS, 2007. 21(6): p. 739-47.

6. Benharrosh, J., et al., [Comparison of two ELISA tests to study the seroprevalence of herpes simplex 1 et 2 infection in a maternity near Paris]. Ann Biol Clin (Paris), 2008. 66(6): p. 665-70.

7. Benjamin, R.J., et al., Human immunodeficiency virus-1 infection correlates strongly with herpes simplex virus-2 (genital herpes) seropositivity in South African and United States blood donations. Transfusion, 2008. 48(2): p. 295-303.

8. Berntsson, M., et al., Decreasing prevalence of herpes simplex virus-2 antibodies in selected groups of women in Sweden. Acta Derm Venereol, 2009. 89(6): p. 623-6.

9. Biswas, D., et al., Seroprevalence and risk factors of herpes simplex virus type-2 infection among pregnant women in Northeast India. BMC Infect Dis, 2011. 11: p. 325.

10. Mahanta, J., et al., Circumcision and herpes simplex virus-2 infection among spouses. Sex Transm Infect, 2010. 86(7): p. 487.

11. Braunstein, S.L., et al., High burden of prevalent and recently acquired HIV among female sex workers and female HIV voluntary testing center clients in Kigali, Rwanda. PLoS One, 2011. 6(9): p. e24321.

12. Brazzale, A.G., et al., Seroprevalence of herpes simplex virus type 1 and type 2 among the Indigenous population of Cape York, Far North Queensland, Australia. Sex Health, 2010. 7(4): p. 453-9.

13. Buka, S.L., et al., Maternal exposure to herpes simplex virus and risk of psychosis among adult offspring. Biol Psychiatry, 2008. 63(8): p. 809-15.

14. Carcamo, C.P., et al., Prevalences of sexually transmitted infections in young adults and female sex workers in Peru: a national population-based survey. Lancet Infect Dis, 2012. 12(10): p. 765-73.

15. Charpentier, C., et al., Distribution of HIV-1 and HSV-2 epidemics in Chad revealing HSV-2 hot-spot in regions of high-risk HIV spread. J Infect Dev Ctries, 2011. 5(1): p. 64-7.

16. Chawla, R., et al., Community-based study on seroprevalence of herpes simplex virus type 2 infection in New Delhi. Indian J Med Microbiol, 2008. 26(1): p. 34-9.

17. Chen, X.S., et al., Herpes simplex virus 2 infection in women attending an antenatal clinic in Fuzhou, China. Sex Transm Infect, 2007. 83(5): p. 369-70.

18. Clark, J.L., et al., Prevalence of HIV, herpes simplex virus-2, and syphilis in male sex partners of pregnant women in Peru. BMC Public Health, 2008. 8: p. 65.

19. Clemens, S.A. and C.K. Farhat, Seroprevalence of herpes simplex 1-2 antibodies in Brazil. Rev Saude Publica, 2010. 44(4): p. 726-34.

20. Cunningham, A.L., et al., Prevalence of infection with herpes simplex virus types 1 and 2 in Australia: a nationwide population based survey. Sex Transm Infect, 2006. 82(2): p. 164-8.

21. Page, A., et al., Upstairs and downstairs: socio-economic and gender interactions in herpes simplex virus type 2 seroprevalence in australia. Sex Transm Dis, 2009. 36(6): p. 344-9.

22. Davidovici, B.B., et al., Decline in the prevalence of antibodies to herpes simplex virus types 1 and 2 among Israeli young adults between 1984 and 2002. Sex Transm Dis, 2006. 33(11): p. 641-5.

23. Davidovici, B.B., et al., Seroprevalence of herpes simplex virus 1 and 2 and correlates of infection in Israel. J Infect, 2006. 52(5): p. 367-73.

24. Davidovici, B.B., et al., Comparison of the dynamics and correlates of transmission of Herpes Simplex Virus-1 (HSV-1) and Varicella-Zoster Virus (VZV) in a sample of the Israeli population. Eur J Epidemiol, 2007. 22(9): p. 641-6.

25. Davies, S.C., et al., Prevalence and risk factors for herpes simplex virus type 2 antibodies among low- and high-risk populations in Indonesia. Sex Transm Dis, 2007. 34(3): p. 132-8.

26. Delany-Moretlwe, S., et al., Comparison of focus HerpesSelect and Kalon HSV-2 gG2 ELISA serological assays to detect herpes simplex virus type 2 antibodies in a South African population. Sex Transm Infect, 2010. 86(1): p. 46-50.

27. Kane, C.T., et al., Concentrated and linked epidemics of both HSV-2 and HIV-1/HIV-2 infections in Senegal: public health impacts of the spread of HIV. Int J STD AIDS, 2009. 20(11): p. 793-6.

28. Diawara, S., et al., Low seroprevalence of herpes simplex virus type 2 among pregnant women in Senegal. Int J STD AIDS, 2008. 19(3): p. 159-60.

29. Dickson, N., et al., Risk of herpes simplex virus type 2 acquisition increases over early adulthood: evidence from a cohort study. Sex Transm Infect, 2007. 83(2): p. 87-90.

30. Doi, Y., et al., Seroprevalence of herpes simplex virus 1 and 2 in a population-based cohort in Japan. J Epidemiol, 2009. 19(2): p. 56-62.

31. Dolar, N., et al., Seroprevalence of herpes simplex virus type 1 and type 2 in Turkey. J Eur Acad Dermatol Venereol, 2006. 20(10): p. 1232-6.

32. Dordevic, H., [Serological response to herpes simplex virus type 1 and 2 infection among women of reproductive age]. Med Pregl, 2006. 59(11-12): p. 591-7.

33. Fife, K.H., et al., Incidence and prevalence of herpes simplex virus infections in adolescent women. Sex Transm Dis, 2006. 33(7): p. 441-4.

34. Ghebremichael, M., D. Habtzgi, and E. Paintsil, Deciphering the epidemic synergy of herpes simplex virus type 2 (HSV-2) on human immunodeficiency virus type 1 (HIV-1) infection among women in sub-Saharan Africa. BMC Res Notes, 2012. 5: p. 451.

35. Ghebremichael, M. and E. Paintsil, High risk behaviors and sexually transmitted infections among men in Tanzania. AIDS Behav, 2011. 15(5): p. 1026-32.

36. Ghebremichael, M., U. Larsen, and E. Paintsil, Association of age at first sex with HIV-1, HSV-2, and other sexual transmitted infections among women in northern Tanzania. Sex Transm Dis, 2009. 36(9): p. 570-6.

37. Glynn, J.R., et al., Herpes simplex virus type 2 trends in relation to the HIV epidemic in northern Malawi. Sex Transm Infect, 2008. 84(5): p. 356-60.

38. Gorander, S., et al., Seroprevalences of herpes simplex virus type 2, five oncogenic human papillomaviruses, and Chlamydia trachomatis in Katowice, Poland. Clin Vaccine Immunol, 2008. 15(4): p. 675-80.

39. Gorander, S., et al., Mature glycoprotein g presents high performance in diagnosing herpes simplex virus type 2 infection in sera of different tanzanian cohorts. Clin Vaccine Immunol, 2006. 13(6): p. 633-9.

40. Tobian, A.A., et al., Factors associated with the prevalence and incidence of herpes simplex virus type 2 infection among men in Rakai, Uganda. J Infect Dis, 2009. 199(7): p. 945-9.

41. Tobian, A.A., et al., Human papillomavirus incidence and clearance among HIV-positive and HIV-negative men in sub-Saharan Africa. AIDS, 2012. 26(12): p. 1555-65.

42. Gray, R.H., et al., Effects of genital ulcer disease and herpes simplex virus type 2 on the efficacy of male circumcision for HIV prevention: Analyses from the Rakai trials. PLoS Med, 2009. 6(11): p. e1000187.

43. Tobian, A.A., et al., Male circumcision for the prevention of HSV-2 and HPV infections and syphilis. N Engl J Med, 2009. 360(13): p. 1298-309.

44. Gutierrez, J.P., et al., Herpes simplex virus type 2 among Mexican high school adolescents: prevalence and association with community characteristics. Arch Med Res, 2007. 38(7): p. 774-82.

45. Haddow, L.J., et al., Herpes simplex virus type 2 (HSV-2) infection in women attending an antenatal clinic in the South Pacific island nation of Vanuatu. Sex Transm Dis, 2007. 34(5): p. 258-61.

46. Hagan, H., et al., Herpes simplex virus type 2 associated with HIV infection among New York heterosexuals living in high-risk areas. Int J STD AIDS, 2010. 21(8): p. 580-3.

47. Heffron, R., et al., High prevalent and incident HIV-1 and herpes simplex virus 2 infection among male migrant and non-migrant sugar farm workers in Zambia. Sex Transm Infect, 2011. 87(4): p. 283-8.

48. Hettmann, A., et al., Seroprevalence of HSV-2 in Hungary and comparison of the HSV-2 prevalence of pregnant and infertile women. Acta Microbiol Immunol Hung, 2008. 55(4): p. 429-36.

49. Jafarzadeh, A., et al., The association between infection burden in Iranian patients with acute myocardial infarction and unstable angina. Acta Med Indones, 2011. 43(2): p. 105-11.

50. Jennings, J.M., et al., Geographic prevalence and multilevel determination of community-level factors associated with herpes simplex virus type 2 infection in Chennai, India. Am J Epidemiol, 2008. 167(12): p. 1495-503.

51. Jewkes, R., et al., Impact of stepping stones on incidence of HIV and HSV-2 and sexual behaviour in rural South Africa: cluster randomised controlled trial. BMJ, 2008. 337: p. a506.

52. Jewkes, R.K., et al., Associations between childhood adversity and depression, substance abuse and HIV and HSV2 incident infections in rural South African youth. Child Abuse Negl, 2010. 34(11): p. 833-41.

53. Jonsson, M.K., et al., Minimal change in HSV-2 seroreactivity: a cross-sectional Swedish population study. Scand J Infect Dis, 2006. 38(5): p. 357-65.

54. Juhl, D., et al., Detection of herpes simplex virus DNA in plasma of patients with primary but not with recurrent infection: implications for transfusion medicine? Transfus Med, 2010. 20(1): p. 38-47.

55. Kasubi, M.J., et al., Prevalence of antibodies against herpes simplex virus types 1 and 2 in children and young people in an urban region in Tanzania. J Clin Microbiol, 2006. 44(8): p. 2801-7.

56. Kaur, R., N. Gupta, and U.K. Baveja, Seroprevalence of HSV1 and HSV2 infections in family planning clinic attenders. J Commun Dis, 2005. 37(4): p. 307-9.

57. Khryanin, A.A. and O.V. Reshetnikov, Seroprevalence of herpes simplex virus type 2 infection in Russia. Int J STD AIDS, 2007. 18(11): p. 797-8.

58. Kim, I.D., H.S. Chang, and K.J. Hwang, Herpes simplex virus 2 infection rate and necessity of screening during pregnancy: a clinical and seroepidemiologic study. Yonsei Med J, 2012. 53(2): p. 401-7.

59. Kirakoya-Samadoulougou, F., et al., Epidemiology of herpes simplex virus type 2 infection in rural and urban Burkina Faso. Sex Transm Dis, 2011. 38(2): p. 117-23.

60. Kirakoya-Samadoulougou, F., et al., Bacterial vaginosis among pregnant women in Burkina Faso. Sex Transm Dis, 2008. 35(12): p. 985-9.

61. Konda, K.A., et al., The epidemiology of herpes simplex virus type 2 infection in low-income urban populations in coastal Peru. Sex Transm Dis, 2005. 32(9): p. 534-41.

62. Kramer, M.A., et al., Ethnic differences in HSV1 and HSV2 seroprevalence in Amsterdam, the Netherlands. Euro Surveill, 2008. 13(24).

63. Kucera, P., et al., Seroepidemiology of herpes simplex virus type 1 and 2 in pregnant women in Switzerland: an obstetric clinic based study. Eur J Obstet Gynecol Reprod Biol, 2012. 160(1): p. 13-7.

64. Le, H.V., et al., Herpes simplex virus type-2 seropositivity among ever married women in South and north Vietnam: a population-based study. Sex Transm Dis, 2009. 36(10): p. 616-20.

65. Li, J.M., et al., Screening of Herpes simplex virus 2 infection among pregnant women in southern China. J Dermatol, 2011. 38(2): p. 120-4.

66. Lin, H., et al., Herpes simplex virus infections among rural residents in eastern China. BMC Infect Dis, 2011. 11: p. 69.

67. Lupi, O., Prevalence and risk factors for herpes simplex infection among patients at high risk for HIV infection in Brazil. Int J Dermatol, 2011. 50(6): p. 709-13.

68. Madhivanan, P., et al., Prevalence of Trichomonas vaginalis infection among young reproductive age women in India: implications for treatment and prevention. Sex Health, 2009. 6(4): p. 339-44.

69. Madhivanan, P., et al., Incidence of herpes simplex virus type 2 in young reproductive age women in Mysore, India. Indian J Pathol Microbiol, 2011. 54(1): p. 96-9.

70. Madhivanan, P., et al., Prevalence and correlates of bacterial vaginosis among young women of reproductive age in Mysore, India. Indian J Med Microbiol, 2008. 26(2): p. 132-7.

71. Mahjour, S.B., et al., Seroprevalence of human herpes simplex, hepatitis B and epstein-barr viruses in children with acute lymphoblastic leukemia in southern iran. Pathol Oncol Res, 2010. 16(4): p. 579-82.

72. Maral, I., et al., Seroprevalences of herpes simplex virus type 2 and Chlamydia trachomatis in Turkey. Arch Gynecol Obstet, 2009. 280(5): p. 739-43.

73. Mehta, S.D., et al., Herpes simplex virus type 2 infection among young uncircumcised men in Kisumu, Kenya. Sex Transm Infect, 2008. 84(1): p. 42-8.

74. Mehta, S.D., et al., Circumcision status and incident herpes simplex virus type 2 infection, genital ulcer disease, and HIV infection. AIDS, 2012. 26(9): p. 1141-9.

75. Mehta, S.D., et al., The effect of medical male circumcision on urogenital Mycoplasma genitalium among men in Kisumu, Kenya. Sex Transm Dis, 2012. 39(4): p. 276-80.

76. Smith, J.S., et al., Increased risk of HIV acquisition among Kenyan men with human papillomavirus infection. J Infect Dis, 2010. 201(11): p. 1677-85.

77. Mir, A.M., et al., Herpes Simplex Virus-2 infection amongst urban male population in Pakistan. J Pak Med Assoc, 2010. 60(11): p. 918-22.

78. Mir, A.M., et al., STI prevalence and associated factors among urban men in Pakistan. Sex Transm Infect, 2009. 85(3): p. 199-200.

79. Miskulin, M., et al., Objective identification of sexual risk behavior among blood donors in Croatia: is it reality? Clin Lab, 2012. 58(1-2): p. 19-25.

80. Miskulin, M., et al., Prevalence and risk factors for herpes simplex virus type 2 infections in East Croatia. Coll Antropol, 2011. 35(1): p. 9-14.

81. Moss, N.J., et al., Predictors of incident herpes simplex virus type 2 infections in young women at risk for unintended pregnancy in San Francisco. BMC Infect Dis, 2007. 7: p. 113.

82. Mowry, E.M., et al., Vitamin D status and antibody levels to common viruses in pediatric-onset multiple sclerosis. Mult Scler, 2011. 17(6): p. 666-71.

83. Mugo, N., et al., Prevalence of herpes simplex virus type 2 infection, human immunodeficiency virus/herpes simplex virus type 2 coinfection, and associated risk factors in a national, population-based survey in Kenya. Sex Transm Dis, 2011. 38(11): p. 1059-66.

84. Mwandi, Z., et al., Male circumcision programmes in Kenya: lessons from the Kenya AIDS Indicator Survey 2007. Bull World Health Organ, 2012. 90(9): p. 642-51.

85. Kimani, D., et al., Blood donors in Kenya: a comparison of voluntary and family replacement donors based on a population-based survey. Vox Sang, 2011. 100(2): p. 212-8.

86. Munjoma, M.W., et al., The prevalence, incidence and risk factors of herpes simplex virus type 2 infection among pregnant Zimbabwean women followed up nine months after childbirth. BMC Womens Health, 2010. 10: p. 2.

87. Munjoma, M.W., M.P. Mapingure, and B. Stray-Pedersen, Risk factors for herpes simplex virus type 2 and its association with HIV among pregnant teenagers in Zimbabwe. Sex Health, 2010. 7(1): p. 87-9.

88. Schillinger, J.A., et al., Seroprevalence of herpes simplex virus type 2 and characteristics associated with undiagnosed infection: New York City, 2004. Sex Transm Dis, 2008. 35(6): p. 599-606.

89. Nguyen, T.Q., et al., Population prevalence of reported and unreported HIV and related behaviors among the household adult population in New York City, 2004. AIDS, 2008. 22(2): p. 281-7.

90. Nilsen, A., et al., Prevalence of, and risk factors for, HSV-2 antibodies in sexually transmitted disease patients, healthy pregnant females, blood donors and medical students in Tanzania and Norway. Epidemiol Infect, 2005. 133(5): p. 915-25.

91. Nyiro, J.U., et al., Seroprevalence, predictors and estimated incidence of maternal and neonatal Herpes Simplex Virus type 2 infection in semi-urban women in Kilifi, Kenya. BMC Infect Dis, 2011. 11: p. 155.

92. Ozdemir, R., et al., [HSV-1 and HSV-2 seropositivity rates in pregnant women admitted to Izmir Ataturk Research and Training Hospital, Turkey]. Mikrobiyol Bul, 2009. 43(4): p. 709-11.

93. Page, W.F., et al., National estimates of seroincidence and seroprevalence for herpes simplex virus type 1 and type 2 among US military adults aged 18 to 29 years. Sex Transm Dis, 2012. 39(4): p. 241-50.

94. Papadogeorgakis, H., et al., Herpes simplex virus seroprevalence among children, adolescents and adults in Greece. Int J STD AIDS, 2008. 19(4): p. 272-8.

95. Patnaik, P., et al., Type-specific seroprevalence of herpes simplex virus type 2 and associated risk factors in middle-aged women from 6 countries: the IARC multicentric study. Sex Transm Dis, 2007. 34(12): p. 1019-24.

96. Patterson, J., et al., Genital HSV detection among HIV-1-infected pregnant women in labor. Infect Dis Obstet Gynecol, 2011. 2011: p. 157680.

97. Reinheimer, C. and H.W. Doerr, Prevalence of herpes simplex virus type 2 in different risk groups: thirty years after the onset of HIV. Intervirology, 2012. 55(6): p. 395-400.

98. Rode, O.D., S.Z. Lepej, and J. Begovac, Seroprevalence of herpes simplex virus type 2 in adult HIV-infected patients and blood donors in Croatia. Coll Antropol, 2008. 32(3): p. 693-5.

99. Sanchez-Aleman, M.A., et al., HSV-2 seroincidence among Mexican college students: the delay of sexual debut is not enough to avoid risky sexual behaviours and virus transmission. Sex Transm Infect, 2010. 86(7): p. 565-9.

100. Sasadeusz, J.J., et al., Prevalence of HSV-2 antibody in a Melbourne antenatal population attending a tertiary obstetric hospital. Aust N Z J Obstet Gynaecol, 2008. 48(3): p. 266-72.

101. Sauerbrei, A., et al., Seroprevalence of herpes simplex virus type 1 and type 2 in Thuringia, Germany, 1999 to 2006. Euro Surveill, 2011. 16(44).

102. Schneider, J.A., et al., Population-based seroprevalence of HSV-2 and syphilis in Andhra Pradesh state of India. BMC Infect Dis, 2010. 10: p. 59.

103. Sgaier, S.K., et al., Prevalence and correlates of Herpes Simplex Virus-2 and syphilis infections in the general population in India. Sex Transm Infect, 2011. 87(2): p. 94-100.

104. Shin, H.S., et al., Herpes simplex virus type 2 seroprevalence in Korea: rapid increase of HSV-2 seroprevalence in the 30s in the southern part. J Korean Med Sci, 2007. 22(6): p. 957-62.

105. Sierra, C.A., et al., Prevalence of specific herpes simplex virus-2 antibodies and associated factors in women of a rural town of Colombia. Trans R Soc Trop Med Hyg, 2011. 105(4): p. 232-8.

106. Smith, J.S., et al., Type specific seroprevalence of HSV-1 and HSV-2 in four geographical regions of Poland. Sex Transm Infect, 2006. 82(2): p. 159-63.

107. Sobngwi-Tambekou, J., et al., Effect of HSV-2 serostatus on acquisition of HIV by young men: results of a longitudinal study in Orange Farm, South Africa. J Infect Dis, 2009. 199(7): p. 958-64.

108. Tarnaud, C., et al., Association of low-risk human papillomavirus infection with male circumcision in young men: results from a longitudinal study conducted in Orange Farm (South Africa). Infect Dis Obstet Gynecol, 2011. 2011: p. 567408.

109. Tedla, Y., et al., Serum antibodies to Toxoplasma gondii and Herpesvidae family viruses in individuals with schizophrenia and bipolar disorder: a case-control study. Ethiop Med J, 2011. 49(3): p. 211-20.

110. Tobian, A.A., et al., Male circumcision and herpes simplex virus type 2 infection in female partners: a randomized trial in Rakai, Uganda. J Infect Dis, 2012. 205(3): p. 486-90.

111. Todd, C.S., et al., Cross-sectional assessment of prevalence and correlates of blood-borne and sexually-transmitted infections among Afghan National Army recruits. BMC Infect Dis, 2012. 12: p. 196.

112. Todd, J., et al., Risk factors influencing HIV infection incidence in a rural African population: a nested case-control study. J Infect Dis, 2006. 193(3): p. 458-66.

113. Vaccarella, S., et al., Sexual behavior, condom use, and human papillomavirus: pooled analysis of the IARC human papillomavirus prevalence surveys. Cancer Epidemiol Biomarkers Prev, 2006. 15(2): p. 326-33.

114. Morrison, C.S., et al., Hormonal contraception and the risk of HIV acquisition. AIDS, 2007. 21(1): p. 85-95.

115. Veldhuijzen, N.J., et al., Prevalence and concordance of HPV, HIV, and HSV-2 in heterosexual couples in Kigali, Rwanda. Sex Transm Dis, 2012. 39(2): p. 128-35.

116. Venkatesh, K.K., et al., African women recently infected with HIV-1 and HSV-2 have increased risk of acquiring Neisseria gonorrhoeae and Chlamydia trachomatis in the Methods for Improving Reproductive Health in Africa trial. Sex Transm Dis, 2011. 38(6): p. 562-70.

117. Venkatesh, K.K., et al., The relative contribution of viral and bacterial sexually transmitted infections on HIV acquisition in southern African women in the Methods for Improving Reproductive Health in Africa study. Int J STD AIDS, 2011. 22(4): p. 218-24.

118. Vilibic-Cavlek, T., et al., Seroprevalence of TORCH infections in women of childbearing age in Croatia. J Matern Fetal Neonatal Med, 2011. 24(2): p. 280-3.

119. Vilibic-Cavlek, T., et al., Herpes simplex virus infection in the Croatian population. Scand J Infect Dis, 2011. 43(11-12): p. 918-22.

120. Watson-Jones, D., et al., Risk factors for herpes simplex virus type 2 and HIV among women at high risk in northwestern Tanzania: preparing for an HSV-2 intervention trial. J Acquir Immune Defic Syndr, 2007. 46(5): p. 631-42.

121. Yahya-Malima, K.I., et al., HIV-1, HSV-2 and syphilis among pregnant women in a rural area of Tanzania: prevalence and risk factors. BMC Infect Dis, 2008. 8: p. 75.

122. Zhu, J., et al., Prevalence and persistence of antibodies to herpes viruses, Chlamydia pneumoniae and Helicobacter pylori in Alaskan Eskimos: the GOCADAN Study. Clin Microbiol Infect, 2006. 12(2): p. 118-22.

123. Xu, F., et al., Trends in herpes simplex virus type 1 and type 2 seroprevalence in the United States. JAMA, 2006. 296(8): p. 964-73.

124. Seroprevalence of herpes simplex virus type 2 among persons aged 14-49 years--United States, 2005-2008. MMWR Morb Mortal Wkly Rep, 2010. 59(15): p. 456-9.

125. Mermin, J., et al., Risk factors for recent HIV Infection in Uganda. JAMA - Journal of the American Medical Association, 2008. 300(5): p. 540-549.

126. Shebl, F.M., et al., Human herpesvirus 8 seropositivity among sexually active adults in Uganda. PLoS One, 2011. 6(6).

127. Ghebremichael, M., E. Paintsil, and U. Larsen, Alcohol abuse, sexual risk behaviors, and sexually transmitted infections in women in moshi Urban district, Northern Tanzania. Sexually Transmitted Diseases, 2009. 36(2): p. 102-107.

128. Kapiga, S.H., et al., The epidemiology of HIV-1 infection in northern Tanzania: Results from a community-based study. AIDS Care - Psychological and Socio-Medical Aspects of AIDS/HIV, 2006. 18(4): p. 379-387.

129. Johnson, K.E., et al., Foreskin inflammation is associated with HIV and herpes simplex virus type-2 infections in Rakai, Uganda. AIDS, 2009. 23(14): p. 1807-1815.

130. Tobian, A.A.R., et al., Incident HIV and herpes simplex virus type 2 infection among men in Rakai, Uganda. AIDS, 2009. 23(12): p. 1589-1594.

131. Panchanadeswaran, S., et al., Gender differences in the prevalence of sexually transmitted infections and genital symptoms in an urban setting in southern India. Sexually Transmitted Infections, 2006. 82(6): p. 491-495.

132. Caceres, C.F., et al., Sexually transmitted disease and HIV prevalence and risk factors in concentrated and generalized HIV epidemic settings. AIDS, 2007. 21(SUPPL. 2): p. S81-S90.

133. Clark, J.L., et al., Prevalence of same-sex sexual behavior and associated characteristics among low-income urban males in Peru. PLoS One, 2007. 2(8).

134. Heiligenberg, M., et al., Seroprevalence and determinants of eight high-risk human papillomavirus types in homosexual men, heterosexual men, and women: A population-based study in Amsterdam. Sexually Transmitted Diseases, 2010. 37(11): p. 672-680.

135. Otieno-Nyunya, B., et al., Epidemiology of syphilis in Kenya: Results from a nationally representative serological survey. Sexually Transmitted Infections, 2011. 87(6): p. 521-525.

136. Cherutich, P., et al., Lack of knowledge of HIV status a major barrier to HIV prevention, care and treatment efforts in Kenya: Results from a nationally representative study. PLoS One, 2012. 7(5).

137. Kurewa, N.E., et al., The burden and risk factors of Sexually Transmitted Infections and Reproductive Tract Infections among pregnant women in Zimbabwe. BMC Infect Dis, 2009. 10(127).

138. Mapingure, M.P., et al., Sexual behaviour does not reflect HIV-1 prevalence differences: A comparison study of Zimbabwe and Tanzania. Journal of the International AIDS Society, 2010. 13(1).

139. Auvert, B., et al., Association of oncogenic and nononcogenic human papillomavirus with HIV incidence. Journal of Acquired Immune Deficiency Syndromes, 2010. 53(1): p. 111-116.

140. Vaccarella, S., et al., Seroprevalence of antibodies against human papillomavirus (HPV) types 16 and 18 in four continents: The International Agency for Research on Cancer HPV prevalence surveys. Cancer Epidemiology Biomarkers and Prevention, 2010. 19(9): p. 2379-2388.

141. Brown, J.M., et al., Incident and prevalent herpes simplex virus type 2 infection increases risk of HIV acquisition among women in Uganda and Zimbabwe. AIDS, 2007. 21(12): p. 1515-1523.

142. Van Der Pol, B., et al., Trichomonas vaginalis infection and human immunodeficiency virus acquisition in African women. Journal of Infectious Diseases, 2008. 197(4): p. 548-554.

143. Morrison, C.S., et al., Pregnancy and the risk of HIV-1 acquisition among women in Uganda and Zimbabwe. AIDS, 2007. 21(8): p. 1027-1034.

144. De Bruyn, G., et al., The effect of the vaginal diaphragm and lubricant gel on acquisition of HSV-2. Sexually Transmitted Infections, 2011. 87(4): p. 301-305.

145. Mavedzenge, S.N., et al., Determinants of differential HIV incidence among women in three southern African locations. Journal of Acquired Immune Deficiency Syndromes, 2011. 58(1): p. 89-99.

146. Wand, H. and G. Ramjee, The relationship between age of coital debut and HIV seroprevalence among women in Durban, South Africa: A cohort study. BMJ Open, 2012. 2(1).

147. Fukuchi, E., et al., Cervical human papillomavirus incidence and persistence in a cohort of HIV-negative women in zimbabwe. Sexually Transmitted Diseases, 2009. 36(5): p. 305-311.

148. Adamson, P.C., et al., Prevalence & correlates of primary infertility among young women in Mysore, India. Indian Journal of Medical Research, 2011. 134(10): p. 440-446.

149. Alberts, C.J., et al., Association of chlamydia trachomatis infection and herpes simplex virus type 2 serostatus with genital human papillomavirus infection in men: The HPV in men study. Sexually Transmitted Diseases, 2013. 40(6): p. 508-515.

150. Amornkul, P.N., et al., HIV prevalence and associated risk factors among individuals aged 13-34 years in rural Western Kenya. PLoS One, 2009. 4(7).

151. Asgari, S., et al., HSV-2 seroepidemiology and risk factors among Iranian women: A time to new thinking. Iranian Red Crescent Medical Journal, 2011. 13(11): p. 818-823.

152. Behanzin, L., et al., Decline in HIV Prevalence among Young Men in the General Population of Cotonou, Benin, 1998-2008. PLoS One, 2012. 7(8).

153. Benzaken, A., et al., HIV and sexually transmitted infections at the borderlands: Situational analysis of sexual health in the Brazilian Amazon. Sexually Transmitted Infections, 2012. 88(4): p. 294-300.

154. Biraro, S., et al., Effect of HSV-2 on population-level trends in HIV incidence in Uganda between 1990 and 2007. Tropical Medicine and International Health, 2013. 18(10): p. 1257-1266.

155. Birdthistle, I.J., et al., From affected to infected? Orphanhood and HIV risk among female adolescents in urban Zimbabwe. AIDS, 2008. 22(6): p. 759-766.

156. Wu, Z., et al., Sexually transmitted diseases and risk behaviors among market vendors in China. Sexually Transmitted Diseases, 2007. 34(12): p. 1030-1034.

157. Chege, W., et al., Baseline findings of an HIV incidence cohort study to prepare for future HIV prevention clinical trials in Kisumu, Kenya. J Infect Dev Ctries, 2012. 6(12): p. 870-880.

158. Cowan, F.M., et al., The Regai Dzive Shiri Project: A cluster randomised controlled trial to determine the effectiveness of a multi-component community-based HIV prevention intervention for rural youth in Zimbabwe - Study design and baseline results. Tropical Medicine and International Health, 2008. 13(10): p. 1235-1244.

159. Pascoe, S.J.S., et al., Increased risk of HIV-infection among school-attending orphans in rural Zimbabwe. AIDS Care - Psychological and Socio-Medical Aspects of AIDS/HIV, 2010. 22(2): p. 206-220.

160. Crucitti, T., et al., Non-sexual transmission of Trichomonas vaginalis in adolescent girls attending school in Ndola, Zambia. PLoS One, 2011. 6(1).

161. De Souza, V.A.U.F., et al., Human herpesvirus-8 infection and oral shedding in Amerindian and non-Amerindian populations in the Brazilian Amazon Region. Journal of Infectious Diseases, 2007. 196(6): p. 844-852.

162. Nascimento, M.C., et al., Seroprevalence of kaposi sarcoma-Associated herpesvirus and other serologic markers in the Brazilian amazon. Emerg Infect Dis, 2009. 15(4): p. 663-667.

163. Ferrand, R.A., et al., Undiagnosed HIV infection among adolescents seeking primary health care in Zimbabwe. Clinical Infectious Diseases, 2010. 51(7): p. 844-851.

164. Goncalez, T.T., et al., Human immunodeficiency virus test-seeking motivation in blood donors, Sao Paulo, Brazil. Vox Sang, 2006. 90(3): p. 170-176.

165. Gray, G.E., et al., Safety and efficacy of the HVTN 503/Phambili Study of a clade-B-based HIV-1 vaccine in South Africa: A double-blind, randomised, placebo-controlled test-of-concept phase 2b study. Lancet Infect Dis, 2011. 11(7): p. 507-515.

166. Gutierrez, J.P., et al., Risk behaviors of 15-21 year olds in Mexico lead to a high prevalence of sexually transmitted infections: Results of a survey in disadvantaged urban areas. BMC Public Health, 2006. 6: p. 11P.

167. He, N., et al., Herpes simplex virus-2 infection in male rural migrants in Shanghai, China. International Journal of STD and AIDS, 2009. 20(2): p. 112-114.

168. Herrera-Ortiz, A., et al., Avidity of antibodies against HSV-2 and risk to neonatal transmission among mexican pregnant women. Infect Dis Obstet Gynecol, 2013. 2013(140142).

169. Huang, W.Y., et al., Sexually transmissible infections and prostate cancer risk. Cancer Epidemiology Biomarkers and Prevention, 2008. 17(9): p. 2374-2381.

170. Janbakhash, A., et al., Seroepidemiology of herpes simplex virus type 2 (HSV2) in HIV infected patients in Kermanshah-Iran. Caspian Journal of Internal Medicine, 2012. 3(4): p. 546-549.

171. Kaiser, R., et al., HIV, syphilis, herpes simplex virus 2, and behavioral surveillance among conflict-affected populations in Yei and Rumbek, southern Sudan. AIDS, 2006. 20(6): p. 942-944.

172. Kapina, M., et al., HIV incidence rates and risk factors for urban women in Zambia: Preparing for a microbicide clinical trial. Sexually Transmitted Diseases, 2009. 36(3): p. 129-133.

173. Krone, B., et al., Common infectious agents in multiple sclerosis: A case-control study in children. Multiple Sclerosis, 2008. 14(1): p. 136-139.

174. Kumar, N., et al., Human herpesvirus 8 genoprevalence in populations at disparate risks of Kaposi's sarcoma. Journal of Medical Virology, 2007. 79(1): p. 52-59.

175. Leyland, B., et al., Serologic detection of herpes simplex virus type 2 antibodies among pregnant women using a point-of-care test from Focus Diagnostics. Journal of Clinical Virology, 2009. 44(2): p. 125-128.

176. Mawak, J.D., et al., Seroprevalence and co-infection of herpes simplex virus type 2 and human immunodeficiency virus in Nigeria. Shiraz E Medical Journal, 2012. 13(1): p. 33-39.

177. Msuya, S.E., et al., Prevalence of sexually transmitted infections among pregnant women with known HIV status in northern Tanzania. Reproductive Health, 2009. 6(1).

178. Msuya, S.E., et al., Decline in HIV prevalence among women of childbearing age in Moshi urban, Tanzania. International Journal of STD and AIDS, 2007. 18(10): p. 680-687.

179. Msuya, S.E., et al., HIV among pregnant women in Moshi Tanzania: The role of sexual behavior, male partner characteristics and sexually transmitted infections. AIDS Research and Therapy, 2006. 3(1).

180. Norris, A.H., A.J. Kitali, and E. Worby, Alcohol and transactional sex: How risky is the mix? Social Science and Medicine, 2009. 69(8): p. 1167-1176.

181. Ozdemir, M., et al., Investigation of viral pathogens during pregnancy in a city region in Turkey [Turkiye'nin bir sehrinde gebelik sirasinda viral patojenlerin arastirilmasi]. Anatolian Journal of Clinical Investigation, 2011. 5(2): p. 78-81.

182. Paz-Bailey, G., et al., High rates of STD and sexual risk behaviors among Garifunas in Honduras. Journal of Acquired Immune Deficiency Syndromes, 2009. 51(SUPPL. 1): p. S26-S34.

183. Pisani, E., et al., Basing policy on evidence: Low HIV, STIs, and risk behaviour in Dili, East Timor argue for more focused interventions. Sexually Transmitted Infections, 2006. 82(1): p. 88-93.

184. Rathore, S., A. Jamwal, and V. Gupta, Herpes simplex virus type 2: Seroprevalence in antenatal women. Indian Journal of Sexually Transmitted Diseases, 2010. 31(1): p. 11-15.

185. Ross, D.A., et al., Biological and behavioural impact of an adolescent sexual health intervention in Tanzania: A community-randomized trial. AIDS, 2007. 21(14): p. 1943-1955.

186. Schensul, S.L., et al., Sexually transmitted infections in men in Mumbai slum communities: The relationship of prevalence to risk behavior. Sexually Transmitted Diseases, 2007. 34(7): p. 444-450.

187. Tayyebi, D. and S. Sharifi, Seroepidemiology of infection with herpes simplex virus types 1 and 2 (HSV1 and HSV2) among asymptomatic university students attending Islamic Azad university of Kazeroun, Southwest of Iran. Iranian Journal of Clinical Infectious Diseases, 2010. 5(2): p. 84-88.

188. Topbas, M., et al., Herpes simplex virus type-2 seroprevalence among adults aged 20-49 in Trabzon [Trabzon ilinde 20-49 yas arasindaki eriskinlerde herpes simpleks virus tip 2 seroprevalansi]. Nobel Medicus, 2012. 8(2): p. 85-90.

189. Uribe-Salas, F., et al., Population-based prevalence of antibodies against herpes simplex virus type 2 and socio-demographic characteristics in Mexico. Trans R Soc Trop Med Hyg, 2009. 103(2): p. 151-158.

190. Wang, H., et al., Antibodies to infectious agents and the positive symptom dimension of subclinical psychosis: The TRAILS study. Schizophr Res, 2011. 129(1): p. 47-51.

191. Zhang, J.F. and W.Y. Zhang, Relationship of cytomegalovirus, Chlamydia pneumoniae and herpes simplex virus type 2 infections with preeclampsia. [Chinese]. National Medical Journal of China, 2012. 92(20): p. 1413-1415.

192. Bunnell, R., et al., HIV transmission risk behavior among HIV-infected adults in Uganda: results of a nationally representative survey. AIDS, 2008. 22(5): p. 617-24.

193. Uma, S., et al., Bacterial vaginosis in women of low socioeconomic status living in slum areas in Chennai, India. Sexual health, 2006. 3(4): p. 297-298.

194. Jewkes, R.K., et al., Intimate partner violence, relationship power inequity, and incidence of HIV infection in young women in South Africa: A cohort study. The Lancet, 2010. 376(9734): p. 41-48.

195. van de Wijgert, J.H., et al., Disentangling contributions of reproductive tract infections to HIV acquisition in African Women. Sex Transm Dis, 2009. 36(6): p. 357-64.

196. Birdthistle, I., et al., Is education the link between orphanhood and HIV/HSV-2 risk among female adolescents in urban Zimbabwe? Soc Sci Med, 2009. 68(10): p. 1810-8.

197. Bernstein, D.I., et al., Epidemiology, clinical presentation, and antibody response to primary infection with herpes simplex virus type 1 and type 2 in young women. Clin Infect Dis, 2013. 56(3): p. 344-51.

198. Kapiga, S.H., et al., The Epidemiology of HIV and HSV-2 Infections among Women Participating in Microbicide and Vaccine Feasibility Studies in Northern Tanzania. PLoS One, 2013. 8(7).

199. Tobian, A.A.R., et al., Herpes simplex virus type-2 assay specificity and male circumcision to reduce herpes simplex virus type-2 acquisition. AIDS, 2013. 27(1): p. 147-149.

200. Dennis, L.K., et al., Sexually transmitted infections and prostate cancer among men in the U.S. military. Cancer Epidemiol Biomarkers Prev, 2009. 18(10): p. 2665-71.

201. Holt, B.Y., et al., Planning STI/HIV prevention among refugees and mobile populations: situation assessment of Sudanese refugees. Disasters, 2003. 27(1): p. 1-15.

202. Detels, R., et al., Sexually transmitted disease prevalence and characteristics of market vendors in eastern China. Sex Transm Dis, 2003. 30(11): p. 803-8.

203. Kasraeian, M., M. Movaseghii, and A.F. Ghiam, Seroepidemiological study of herpes simplex virus type 2 (HSV-2) antibody in Shiraz, Iran. Iran J Immunol, 2004. 1(3): p. 189-93.

204. Rezza, G., et al., Human herpesvirus-8 and other viral infections, Papua New Guinea. Emerg Infect Dis, 2001. 7(5): p. 893-5.

205. Pourmand, D., et al., [Seroepidemiological study of herpes simplex virus in pregnant women referring to health and care center in Kermanshah (2003-2004)]. Behbood, 2008. 11(4): p. 462-9.

206. Rotermann, M., et al., Prevalence of Chlamydia trachomatis and herpes simplex virus type 2: results from the 2009 to 2011 Canadian Health Measures Survey. Health Rep, 2013. 24(4): p. 10-5.

207. Howard M., Sellors J.W., Jang D., et al. Regional distribution of antibodies to herpes simplex virus type 1 (HSV-1) and HSV-2 in men and women in Ontario, Canada. J Clin Microbiol, 2003. 41(1): p. 84-9.

208. Leone P., Fleming D.T., Gilsenan A.W., Li L., Justus S. Seroprevalence of herpes simplex virus-2 in suburban primary care offices in the United States. Sex Transm Dis, 2004. 31(5): p. 311-6.

209. Cowan F.M., French R.S., Mayaud P., et al. Seroepidemiological study of herpes simplex virus types 1 and 2 in Brazil, Estonia, India, Morocco, and Sri Lanka. Sex Transm Infect, 2003. 79(4): p. 286-90.

210. Sanchez-Aleman M.A., Conde-Glez C.J., Gayet C., Garcia-Cisneros S., Uribe-Salas F. Sexual behavior and herpes simplex virus 2 infection in college students. Archives of Medical Research, 2005. 36(5): p. 574-580.

211. Abraham C.D., Conde-Glez C.J., Cruz-Valdez A., Sanchez-Zamorano L., Hernandez-Marquez C., Lazcano-Ponce E. Sexual and demographic risk factors for herpes simplex virus type 2 according to schooling level among Mexican youths. Sex Transm Dis, 2003. 30(7): p. 549-55.

212. Smith J.S., Herrero R., Bosetti C., et al. Herpes simplex virus-2 as a human papillomavirus cofactor in the etiology of invasive cervical cancer. J Natl Cancer Inst, 2002. 94(21): p. 1604-13.

213. Abuharfeil N. and Meqdam M.M. Seroepidemiologic study of herpes simplex virus type 2 and cytomegalovirus among young adults in northern Jordan. New Microbiol, 2000. 23: p. 235-9.

214. Dan M., Sadan O., Glezerman M., Raveh D., Samra Z. Prevalence and risk factors for herpes simplex virus type 2 infection among pregnant women in Israel. Sex Transm Dis, 2003. 30(11): p. 835-8.

215. Lagarde E., Congo Z., Meda N., et al. Epidemiology of HIV infection in urban Burkina Faso. Int J STD AIDS, 2004. 15(6): p. 395-402.

216. Ndjoyi-Mbiguino A., Ozouaki F., Legoff J., et al. Comparison of washing and swabbing procedures for collecting genital fluids to assess cervicovaginal shedding of herpes simplex virus type 2 DNA. J Clin Microbiol, 2003. 41(6): p. 2662-4.

217. Hogrefe W., Su X., Song J., Ashley R., Kong L. Detection of herpes simplex virus type 2-specific immunoglobulin G antibodies in African sera by using recombinant gG2, Western blotting, and gG2 inhibition. J Clin Microbiol, 2002. 40(10): p. 3635-40.

218. Pebody R.G., Andrews N., Brown D., et al. The seroepidemiology of herpes simplex virus type 1 and 2 in Europe. Sex Transm Infect, 2004. 80(3): p. 185-91.

219. Alanen A., Kahala K., Vahlberg T., Koskela P., Vainionpaa R. Seroprevalence, incidence of prenatal infections and reliability of maternal history of varicella zoster virus, cytomegalovirus, herpes simplex virus and parvovirus B19 infection in South-Western Finland. Bjog, 2005. 112(1): p. 50-6.

220. Le Donne M., Mancuso A., Leonardi M.S., et al. Sero-prevalence of cytomegalovirus, rubella, herpes simplex virus, varicella zoster virus, measles, parvovirus B19 and adenovirus in women with spontaneous abortion. Italian Journal of Gynaecology & Obstetrics, 2005. 17(1): p. 29-35.

221. Kim O., Kim S.S., Park M.S., et al. Seroprevalence of sexually transmitted viruses in Korean populations including HIV-seropositive individuals. Int J STD AIDS, 2003. 14(1): p. 46-49.

222. Suligoi B., Danaya R.T., Sarmati L., et al. Infection with human immunodeficiency virus, herpes simplex virus type 2, and human herpes virus 8 in remote villages of southwestern Papua New Guinea. Am J Trop Med Hyg, 2005. 72(1): p. 33-6.
